# Rational Design of Mixed Matrix Membranes Modulated by Trisilver Complex for Efficient Propylene/Propane Separation

**DOI:** 10.1002/advs.202206858

**Published:** 2023-02-07

**Authors:** Shenzhen Cong, Xiaoquan Feng, Lili Guo, Donglai Peng, Jing Wang, Jinghuo Chen, Yatao Zhang, Xiangjian Shen, Guang Yang

**Affiliations:** ^1^ School of Chemical Engineering Zhengzhou University Zhengzhou 450001 China; ^2^ College of Chemistry Zhengzhou University Zhengzhou 450001 China; ^3^ School of Material & Chemical Engineering Zhengzhou University of Light Industry Zhengzhou 450001 China

**Keywords:** C_3_H_6_/C_3_H_8_ separation, facilitated transport, mixed matrix membrane, trisilver complex

## Abstract

The application of membrane‐based separation processes for propylene/propane (C_3_H_6_/C_3_H_8_) is extremely promising and attractive as it is poised to reduce the high operation cost of the established low temperature distillation process, but major challenges remain in achieving high gas selectivity/permeability and long‐term membrane stability. Herein, a C_3_H_6_ facilitated transport membrane using trisilver pyrazolate (Ag_3_pz_3_) as a carrier filler is reported, which is uniformly dispersed in a polymer of intrinsic microporosity (PIM‐1) matrix at the molecular level (≈15 nm), verified by several analytical techniques, including 3D‐reconstructed focused ion beam scanning electron microscropy (FIB–SEM) tomography. The *π*‐acidic Ag_3_pz_3_ combines preferentially with *π*‐basic C_3_H_6_, which is confirmed by density functional theory calculations showing that the silver ions in Ag_3_pz_3_ form a reversible *π* complex with C_3_H_6_, endowing the membranes with superior C_3_H_6_ affinity. The resulting membranes exhibit superior stability, C_3_H_6_/C_3_H_8_ selectivity as high as ≈200 and excellent C_3_H_6_ permeability of 306 Barrer, surpassing the upper bound selectivity/permeability performance line of polymeric membranes. This work provides a conceptually new approach of using coordinatively unsaturated 0D complexes as fillers in mixed matrix membranes, which can accomplish olefin/alkane separation with high performance.

## Graphical abstract

A novel trisilver complex with strong *π*‐acidity for coordination with olefins is successfully used to design a well‐dispersed mixed matrix membrane for efficient propylene/propane separation.

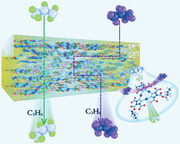

## 1 Introduction

Separation of propylene/propane (C_3_H_6_/C_3_H_8_) mixture represents a class of the most important and also the costliest olefin/paraffin separations in the chemical and petrochemical industry.^[^ ^1^ ^]^ C_3_H_6_ is a prime olefin feedstock for petrochemical production and an essential building block for the manufacture of various chemicals, including polypropylene. The end use of C_3_H_6_ is governed by its purity, because high purity C_3_H_6_ (minimum 99.5 wt%, polymer‐grade specifications) is required for polymer production.^[^ ^2^ ^]^ The purity of C_3_H_6_ primarily depends on the removal of C_3_H_8_, with which it is mixed. As the key physico‐chemical properties of C_3_H_6_ and C_3_H_8_ such as boiling point and molecular size are largely indistinguishable, highly energy‐intensive multiplate low temperature distillation is currently employed industrially to separate the mixture. It is reported that the low temperature separation of 1 ton propylene (>99%) from a propylene/propane mixture derived from steam cracking consumes ≈8.0 × 10^6^ kJ energy, which amounts to 0.3% of the total global energy use for this petrochemical separation every year.^[^ ^3^, ^4^ ^]^ In 2016, one estimate of the global propylene production was 99 million tons and the expected demand growth rate, until 2025, is 4.0%/year.^[^ ^5^ ^]^ The large capital expense and energy cost required for low temperature distillation have aroused extensive research interest for C_3_H_6_/C_3_H_8_ separation by alternative means.^[^ ^6^ ^]^ Membrane‐based processes have been suggested as a way to replace or integrate low temperature distillation units while also lowering energy costs in the separation section.^[^ ^7^, ^8^, ^9^, ^10^, ^11^, ^12^, ^13^ ^]^ It is estimated that the energy consumption of membrane separation for C_3_H_6_/C_3_H_8_ is only 40% that of low temperature distillation.^[^ ^5^ ^]^ Therefore, research on more efficient C_3_H_6_/C_3_H_8_ separation technology is of great value as it has the potential to reduce world energy consumption.

Membrane‐based separation process has produced very encouraging results, specifically in terms of olefin/paraffin separations.^[^ ^14^, ^15^, ^16^, ^17^, ^18^ ^]^ Recently, Lee et al.^[^ ^19^ ^]^ found that a membrane with 11.3 GPU (1 GPU = 1 × 10^−6^ cm^3^ (STP)/cm^2^ s cmHg) of C_3_H_6_ permeability and a C_3_H_6_/C_3_H_8_ selectivity of 68 components is needed to replace a typical C3 distillation separation process. The greatest challenges for membrane technology are to achieve suitably high selectivity/permeability and long‐term operational stability because it is technically and practically feasible to replace the distillation column with membrane units that perform appropriately. However, the harsh operational conditions that membrane must withstand could result in performance loss.^[^ ^20^, ^21^ ^]^

For the separation of C_3_H_6_/C_3_H_8_ and other gas pairs, the permselectivity of pure polymeric membranes are often limited by the selectivity/permeability trade‐off effect. Zeolites and carbon molecular sieve membranes face other difficulties in terms of economical and scalable fabrication, because they are inherently brittle.^[^ ^22^, ^23^ ^]^ On the other hand, silver salt‐doped facilitated transport membranes have been extensively studied and have demonstrated significant achievements for useful C_3_H_6_/C_3_H_8_ selectivity.^[^ ^5^ ^]^ Through reversible interactions (*π*‐complexation) between Ag ions and C_3_H_6_, Ag ions acting as carriers can realize the efficient transport of C_3_H_6_ through the membranes. However, silver‐facilitated transport membranes have shown problems with long‐term stability, owing to the easy poisoning of Ag ions by impurities in the feed stream.^[^ ^24^, ^25^, ^26^ ^]^

Mixed‐matrix membranes (MMMs) are fabricated by dispersing selective fillers in processable polymer matrices, and are an effective strategy for improving the performance of membranes.^[^ ^27^ ^]^ This strategy allows fabrication of a membrane with both enhanced separation performance and scalable fabrication, similar to that for simple, pure polymeric membranes.^[^ ^28^ ^]^ In general, and for C_3_H_6_/C_3_H_8_ separation, MMMs require that the filler should have good compatibility with the polymer matrix, to minimize interfacial defects and nonideal structures. Chung et al. report a series of homogeneous nanocomposite membranes combined with sulfocalixarenes (SCAs) by molecular level dispersion.^[^ ^29^, ^30^ ^]^ The MMMs interfacial compatibility was significantly enhanced. Furthermore, strong interactions with C_3_H_6_ and/or appropriate filler pore size for molecular sieving of C_3_H_6_ from C_3_H_8_ are important. Facilitated membrane transport is achieved by a reversible chemical reaction or complexation combined with a diffusion process, driven by pressure. Typical reversible complexation processes include hydrogen‐bonding, acid–base interactions, chelation, clathration, and *π* bonding.^[^ ^21^ ^]^ A majority of studies have focused on the direct utilization of silver salts as fillers for the preparation of MMMs, whereby porous polymers are frequently used as carriers; an example is AgBF_4_/PEO.^[^ ^5^, ^31^, ^32^ ^]^ Facilitated transport membranes based on Ag^+^ have shown promise in propylene/propane separation, but the long‐term stability of Ag^+^ has precluded industrial adoption.^[^ ^17^, ^33^ ^]^

Here we report facilitated C_3_H_6_ transport membranes based on a triangular trisilver metal ion pyrazolato complex (Ag_3_pz_3_, pz: pyrazole/pyrazole derivatives) through the MMM approach. Ag_3_pz_3_ has *π*‐acidity and thus is expected to selectively combine with *π*‐basic C_3_H_6_,^[^ ^34^, ^35^ ^]^ but this conceptual approach has not yet been explored in the field of membrane separation. Unlike MOFs, Ag_3_pz_3_ is a 0D molecule (≈10 Å) that is soluble in many organic solvents, making it possible to prepare membrane with a molecular‐level dispersion of these fillers.^[^ ^36^ ^]^ In addition, the Ag_3_pz_3_ complex can be tailored to optimize *π*‐acidity and compatibility with the polymeric matrix. Therefore, Ag_3_pz_3_ is a promising material for fabricating C_3_H_6_/C_3_H_8_ separation membranes.

**Figure 1:**
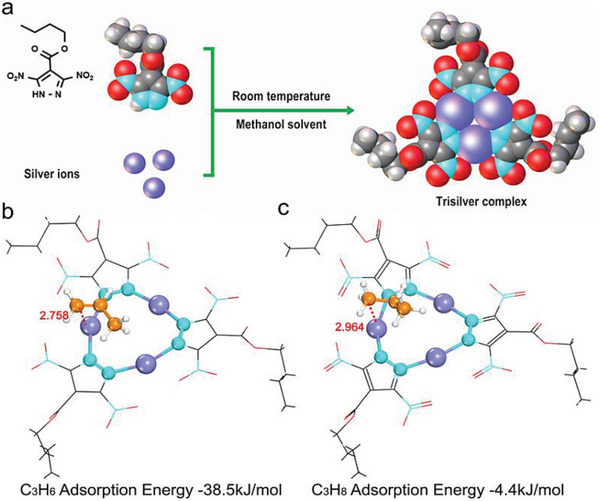
Schematic diagram of the DFT optimized C_3_H_6_ and C_3_H_8_ in Ag_3_pz_3_. a) The synthesis of Ag_3_pz_3_ complex. b) The C_3_H_6_ adsorption energy during the transport process is −38.5 kJ mol_−1_. c) The C_3_H_8_ adsorption energy during the transport process is −4.4 kJ mol^−1^. (The distance is in Angstrom (Å))

Here, we fabricate a new type of facilitated transport membrane for C_3_H_6_/C_3_H_8_ separation, derived from an Ag_3_pz_3_ complex with strong *π*‐acidity. To achieve simultaneously strong *π*‐acidity and good compatibility with the polymer matrix, a pyrazole (butyl 3,5‐dinitro‐1*H*‐pyrazole‐4‐carboxylate) with two electron‐withdrawing nitro‐ and one *n*‐butyl group is designed (Scheme S1, Supporting Information). The Ag^+^ in the complex can form a reversible *π* complex with C_3_H_6_ to facilitate C_3_H_6_ transport and achieve an efficient separation for the C_3_H_6_/C_3_H_8_ (**Figure** **1**). Polymer of intrinsic microporosity (PIMs) contain both an internal through‐hole microporous structure and a high specific surface area of the microporous material, as well as strong thermal stability and solvent processability of the general polymer material.^[^ ^37^, ^38^ ^]^ The molecular structure of PIMs determines its microporous structure, which is unaffected by heat treatment or processing. PIMs' molecular self‐distortion and rigid molecular structure hinders the effective stacking of molecular chains formed an inherent microporous structure between molecular chains.^[^ ^39^, ^40^ ^]^ As a result, PIM‐1 was chosen as the polymer matrix for the production of facilitated transported membrane.

## 2 The Fabrication of Ag_3_pz_3_

Ag_3_pz_3_ was synthesized from butyl‐3,5‐dinitro‐1*H*‐pyrazole‐4‐carboxylate and AgNO_3_ in methanol solvent at ambient temperature for 2 h (Figure 1a), which was characterized by FTIR, ^13^C NMR and ^1^H NMR and powder XRD (Figures S3–S5 and Table S1, Supporting Information). X‐ray analysis reveals that Ag_3_pz_3_ consists of three linear two‐coordinated silver atoms, each pair of which is bridged by a pyrazolyl ligand, forming a planar nine‐membered Ag_3_N_6_ ring (Figure S6, Supporting Information). The presence of strong electron‐withdrawing nitro and ester groups on pyrazolyl ring enhances the *π*‐acidity of Ag^+^, thus favoring the complexation between the Ag_3_pz_3_ and C_3_H_6_.^[^ ^24^ ^]^

To verify the complexing ability of *π*‐acidic Ag^+^ in the Ag_3_pz_3_ complex with C_3_H_6_ and C_3_H_8_, the possible adsorption configurations between Ag_3_pz_3_, C_3_H_6_, and C_3_H_8_, as well as their corresponding adsorption energies were calculated by density functional theory (DFT) (the DFT calculation method is given in the Supporting Information). First, the optimized structural information (Figure S7, Supporting Information) obtained from the DFT calculations is consistent with the experimental crystal structure (Figure S6, Supporting Information). Bader charge analysis results (Figure S8, Supporting Information) show that the Ag^+^ in Ag_3_pz_3_ is an important adsorption site with an average of about 0.6 donor electrons. The adsorption energies of C_3_H_6_ and C_3_H_8_ are −38.5 and −4.4 kJ mol^−1^, respectively, which are about an order of magnitude different. The interatomic distances of the adsorption molecules are illustrated in Figure 1b,c. The distances between the C_3_H_6_ and the single Ag^+^ are distinctly shorter than those of C_3_H_8_ because of the strong *π* complexing, thus confirming that Ag_3_pz_3_ adsorbs C_3_H_6_ much more readily than C_3_H_8_. In addition, DFT calculation also suggests that the center of the triangular Ag_3_pz_3_ molecule could be another adsorption site for C_3_H_6_/C_3_H_8_ separation, although less efficient (Figure S9, Supporting Information). Based on the DFT results, we propose possible transport mechanisms of the membrane: one main facilitating transport process for the reversible acid–base complexation of C_3_H_6_ via a *π*‐acidic single silver of Ag_3_pz_3_, and one synergistic transport process for the reversible acid–base complexation of C_3_H_6_ with the *π*‐acidic Ag_3_pz_3_.

## 3 The Prepartion of Facilitated Transport Membrane

**Figure 2:**
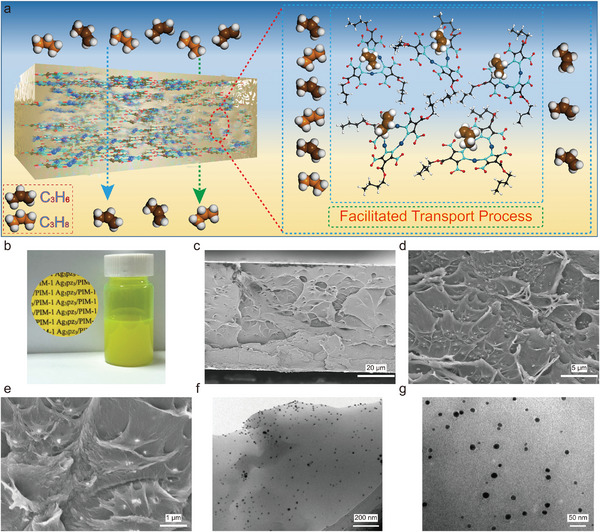
Design and fabrication of the Ag_3_pz_3_/PIM‐1 mixed matrix membrane: a) Schematic of the Ag_3_pz_3_/PIM‐1 facilitated transport membrane (the silver complex facilitates the transport of propylene and endows the membrane with high C_3_H_6_/C_3_H_8_ separation efficiency); b) Optical pictures of Ag_3_pz_3_/PIM‐1‐10 membrane and its casting solution; c–e) Cross‐sectional SEM images of Ag_3_pz_3_/PIM‐1‐10 membrane at various magnifications; f,g) Cross‐sectional TEM images of the Ag_3_pz_3_/PIM‐1‐10 membrane with different magnifications (The inset is the selected area electron diffraction (SAED) of the Ag_3_pz_3_/PIM‐1‐10 membrane).

The facilitated C_3_H_6_ transport membranes are prepared by incorporating Ag_3_pz_3_ nanocrystals into a microporous polymer matrix, polymer of intrinsic microporosity (PIM‐1).^[^ ^41^, ^42^ ^]^ The rigid ladder‐like polymer chain has sites of contortion, endowing PIM‐1 with microporosity, which result in high gas permeability and moderate selectivity. The butyl group on the pyrazolyl ring of Ag_3_pz_3_ serves to improve the solubility of the Ag_3_pz_3_ complex in polymer matrix and organic solvent.^[^ ^43^ ^]^ Apart from solubilizing Ag_3_pz_3_ in organic solvents, the *n*‐butyl groups enhance the compatibility between Ag_3_pz_3_ and the PIM‐1 matrix, effectively minimizing interface defects in the Ag_3_pz_3_/PIM‐1 membrane. As shown in **Figure** **2**d,e and Figure S11 (Supporting Information), the Ag_3_pz_3_ exhibits as a good dispersion in the PIM‐1 polymer matrix with a visually defect‐free Ag_3_pz_3_/PIM‐1 interface. Cross‐sectional TEM images of Ag_3_pz_3_/PIM‐1‐10 membrane indicate that the size of the Ag_3_pz_3_ complex is approximately 15 nm (Figure 2f,g; Figure S12, Supporting Information), and it achieves a molecular level dispersion in the PIM‐1 polymer matrix. The SAED of the Ag_3_pz_3_/PIM‐1‐10 membrane confirms that the structure of the Ag_3_pz_3_ complex retains integrity in the polymer. The chemical features of the PIM‐1 and Ag_3_pz_3_/PIM‐1 facilitated transport membrane is also verified by XRD and FTIR (Figure S11, Supporting Information).

**Figure 3:**
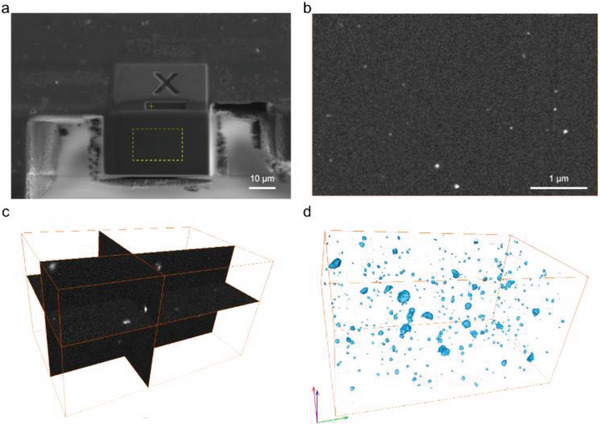
Tomographic FIB–SEM analysis of Ag_3_pz_3_/PIM‐1 mixed matrix membrane: a) Overview SEM image of a focused ion beam (FIB) carved trench on the surface of an Ag_3_pz_3_/PIM‐1‐10 membrane (The central area of the imaged cross‐section that was chosen for additional analysis is indicated by the yellow frame), b) The Ag_3_pz_3_/PIM‐1‐10 membrane’ cross‐sectional SEM 160th piece picture, c) 3D reconstruction of the Ag_3_pz_3_/PIM‐1‐10 membrane’ FIB‐SEM tomogram, d) The Ag_3_pz_3_/PIM‐1‐10 membrane’ segmented FIB‐SEM tomograms (Ag_3_pz_3_ particles are depicted in blue and the boxes' dimensions in the Figure 3d are 5.1:3.2:2.8 m in the *x*, *y*, and *z* directions).

To further explore the structural and physicochemical properties of the Ag_3_pz_3_ complex, alongside their dispersibility in the polymer matrix, which present promising features for their integration into advanced membrane materials for olefin/alkane separation, the internal structure of the Ag_3_pz_3_/PIM‐1 membrane was further analyzed. To investigate the internal structure of the Ag3pz3/PIM‐1‐10 membrane, 3D reconstruction and tomographic FIB‐SEM were used,^[^ ^44^, ^45^ ^]^ as shown in **Figure** **3**. A groove at the observation site was engraved by focused ion beam (FIB) on the upper membrane surface (Figure 3a) and a series of cross‐section SEM images were captured during the continuous FIB milling of thin slices (Figure 3b; Figure S13, Supporting Information). SEM cross‐sectional images show that Ag_3_pz_3_ complex is uniformly dispersed in the polymer matrix, which is consistent with the TEM results of ultrathin sections of the membrane. After aligning the SEM images obtained from the FIB sections, the imaged volumes were reconstructed in 3D. A complete tomogram is provided as Movie S1 (Supporting Information), while surface‐rendered views that have been divided into various phases are shown in Figure 3d and Figure S13 in the Supporting Information. From the results of the 3D reconstruction, the Ag_3_pz_3_ complex exhibited excellent dispersibility in the polymer. The volume size distribution results show that the volume of Ag_3_pz_3_ is ≈500 nm^3^ and the size distribution is relatively uniform (Figure S14, Supporting Information). UV–vis spectroscopy shows that Ag_3_pz_3_/PIM‐1 membranes have a broadband absorption between 200 and 300 nm. The wavelength ranges of absorption bands for different Ag_3_pz_3_/PIM‐1 membranes are listed in Table S4 (Supporting Information), which show that the number of absorption bands decline with decreasing Ag_3_pz_3_ content, with an accompanying blue shift.^[^ ^46^ ^]^ The evident blue shift indicates the quantum size effect of the nano‐material, further verifying the small size of Ag_3_pz_3_ complex in the PIM‐1 matrix.^[^ ^47^ ^]^ The Ag_3_pz_3_ size in the Ag_3_pz_3_/PIM‐1‐20 membrane becomes wider as the content of Ag_3_pz_3_ complex increases (Figure S12, Supporting Information) and this phenomenon is consistent with the quantum size effect.

## 4 The Obtained Membrane Separation Performance

**Figure 4:**
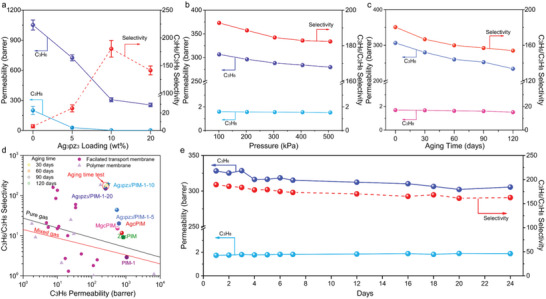
Separation performance of Ag_3_pz_3_/PIM‐1 mixed matrix membrane: a) Single gas C_3_H_6_ permeability and C_3_H_6_/C_3_H_8_ selectivity correlated with various loadings (wt%) of Ag_3_pz_3_ complex in the membrane; b) The effect of temperature and pressure on C_3_H_6_/C_3_H_8_ permselectivity; c) Single gas C_3_H_6,_ C_3_H_8_ permeability and ideal C_3_H_6_/C_3_H_8_ selectivity dependence with aging time of Ag_3_pz_3_/PIM‐1‐10 membrane; d) The performance benchmark based on the state‐of‐the‐art membranes for C_3_H_6_/C_3_H_8_ separation.^[^ ^40^, ^48^ ^]^ The benchmark data were listed in Tables S5, S6, S7, and S8 in the Supporting Information. The solid triangular symbols indicate polymeric membranes, whereas the solid circle symbols represent facilitated transport membranes; e) Long‐term stability of Ag_3_pz_3_/PIM‐1‐10 membrane under single gas conditions.

**Figure 5:**
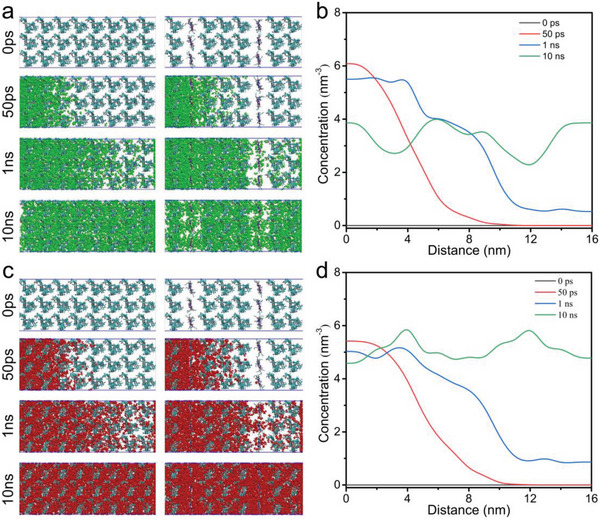
Molecular dynamics (MD) simulation of C_3_H_6_ and C_3_H_8_ diffusion in membrane. Conformation diagram of a) C_3_H_8_ diffusion movement and c) C_3_H_6_ diffusion movement (c) at different moments (left PIM‐1, right Ag_3_pz_3_/PIM‐1). Concentration distribution diagram of b) C_3_H_8_ and d) C_3_H_6_ in Ag_3_pz_3_/PIM‐1 at different times.

Pure C_3_H_6_ and C_3_H_8_ gas permeation on PIM‐1 membranes with Ag_3_pz_3_ different wt% loadings, shown in **Figure** **4**a, demonstrates that selectivity is greatly improved by the incorporation of Ag_3_pz_3_ complex. The permeability of C_3_H_6_ steadily decreases with increasing loading of Ag_3_pz_3_. This trend is the result of an acid–base reversible complexation process between *π*‐acidic Ag^+^ in Ag_3_pz_3_ and *π*‐basic C_3_H_6_. The interaction mechanism between the C_3_H_6_ and Ag^+^ called *π*‐bond complexation.^[^ ^10^ ^]^ The *π*‐bond complexation occurs when the bonding orbital of C_3_H_6_ contributes electronic density to the vacant outermost orbital of Ag^+^ (5s), resulting in the formation of a *σ* bond. The second link created is a *π* bond established by the backdonation of the electronic density from the outermost atomic orbital 4d, which is electrically completed, to the C_3_H_6_’s π*‐ antibonding molecular orbital.^[^ ^5^, ^49^ ^]^ With increasing loading of Ag_3_pz_3_, *π*‐acidic Ag^+^ has multiple acid–base complexation steps with *π*‐basic C_3_H_6_ permeating in the membrane, thus decreasing the permeability of C_3_H_6_. However, compared with C_3_H_6_, the permeability of C_3_H_8_ is reduced more sharply. This may be attributed to two factors: One is that the dispersed Ag_3_pz_3_ complex (*n*‐butyl groups) may serve as a chain stiffener, stiffening the contorted ladder‐like chains and further inducing inefficient chain packing of the PIM‐1 matrix.^[^ ^43^, ^50^ ^]^ The other is that *π*‐acidic Ag^+^ does not complex with C_3_H_8_, thus requiring the C_3_H_8_ to bypass the Ag_3_pz_3_ and permeate through the PIM‐1 matrix (Figure 2a). As the loading of the Ag_3_pz_3_ complex increases, the permeability of C_3_H_8_ tends to stabilize. This is due to a combination of a longer pathway of C_3_H_8_ needed to bypass the complex, and a fewer number of bypassed complexes (the quantum size effect of the complex, i.e., as the content of the Ag_3_pz_3_ complex increases, the size of the Ag_3_pz_3_ complex increases). The mixed gas (C_3_H_6_/C_3_H_8_ 50:50) separation performance was evaluated (Figure 4d). Compared with the pure gas separation performance, the mixed gas performance of the membrane decreased and the gas permeability increased, which indicated that the C_3_H_6_ transport the membrane was affected by other gases. In addition, the presence of C_3_H_8_ reduces the complexing ability of Ag^+^ to C_3_H_6_, thereby reducing the selectivity and increasing the permeability. Compared with bare PIM‐1 membrane, molecular dynamics simulation results show that the diffusion rate of C_3_H_6_ is increased, while the diffusion rate of C_3_H_8_ decreases in Ag_3_pz_3_/PIM‐1 (**Figure** **5**; Figure S20, Supporting Information), which indicates that Ag_3_pz_3_ complex can effectively achieve the separation of C_3_H_6_/C_3_H_8_. The C_3_H_6_ and C_3_H_8_ sorption performance of the membranes was evaluated at 298 K. As shown in Figure S18 (Supporting Information), when compared to the bare PIM‐1 membrane's adsorption capacity to C_3_H_6_ and C_3_H_8_, the adsorption capacity of the Ag_3_pz_3_/PIM‐1‐x hybrid membrane to C_3_H_6_ and C_3_H_8_ is decreased with the Ag_3_pz_3_ loaded. The *n*‐butyl group of Ag_3_pz_3_ fills the pores and reduces the porosity and polymer chain spacing. Despite the complexation of Ag^+^ and C_3_H_6_, the total pore volume and specific surface area of the membrane is reduced (Figure S17, Supporting Information) resulting in fewer adsorption sites and a reduction in C_3_H_6_ adsorption capacity.

To evaluate the potential of the membranes for industrial applications, the gas separation performance of Ag_3_pz_3_/PIM‐1‐10 membrane was investigated at different pressures and temperatures. Figure 4b shows that the C_3_H_6_ permeability decreases with increasing feed gas pressure, which may be caused by membrane plasticization. To further explore the effect of temperature on the reversible acid–base complexation process, the performance of Ag_3_pz_3_/PIM‐1‐10 at different temperatures was evaluated. The C_3_H_6_ permeability and the C_3_H_6_/C_3_H_8_ selectivity decrease with increasing temperature. (Figure S19, Supporting Information). Since the acid–base complexation is an exothermic process, this results in reduced C_3_H_6_ permeability at higher temperatures. For C_3_H_8_, there is a minimal temperature effect on permeability and thus, the C_3_H_6_/C_3_H_8_ selectivity decreases.

The aging behavior of Ag_3_pz_3_/PIM‐1‐10 was evaluated on a membrane stored in an ambient environment. Permeability of C_3_H_6_ initially dropped sharply during the first 60 d as the polymer segments underwent densification, and then the permeability gradually stabilized with extended time. Ag_3_pz_3_ has only a weak complexation with C_3_H_8_ in the facilitated transport process, and concomitantly reduces the permeability of C_3_H_8_ in the polymer matrix. Different from C_3_H_6_, the aging process of the Ag_3_pz_3_/PIM‐1‐10 membrane does not exert a significant effect on the permeability of C_3_H_8_. Therefore, the C_3_H_6_/C_3_H_8_ selectivity of the Ag_3_pz_3_/PIM‐1 membrane tends to remain stable in a long term after the initial decline.

To investigate the stability of Ag_3_pz_3_ in membranes after the long‐term stability test, the Ag_3_pz_3_ in the tested Ag_3_pz_3_/PIM‐1 membrane was collected (the detailed procedure is provided in the Supporting Information). The obtained post‐aging Ag_3_pz_3_ powder was dispersed in CD_3_CN for evaluation by ^1^H NMR spectroscopy (Figures S21 and S22, Supporting Information), and the structure of Ag_3_pz_3_ remained identical. Furthermore, subjecting Ag_3_pz_3_ to an ambient environment for one week resulted in no color change (Figure S23, Supporting Information), clearly showing that the stability of the Ag_3_pz_3_ complex is improved compared with silver(I) salts. In comparison, the silver salt has a visible color change. The long‐term stability of the Ag_3_pz_3_/PIM‐1 membrane was also evaluated for its suitability for industrial application (Figure 4e). The facilitated transport membranes have greater selectivity for olefin purification than some reported solids‐facilitated transport membranes and ionic liquid membranes (Figure S8, Supporting Information). In addition, comparing with some reported solids‐facilitated transport membranes, the Ag_3_pz_3_/PIM‐1 membrane has better long‐term stability (Tables S6 and S7, Supporting Information).

The C_3_H_6_/C_3_H_8_ separation performance of the facilitated transport membrane far surpasses the upper bound for polymeric membranes (Figure 4d).^[^ ^48^ ^]^ Compared with polymeric membranes and mixed‐matrix membranes in previous studies, the C_3_H_6_/C_3_H_8_ separation performance of Ag_3_pz_3_/PIM‐1 membrane is significantly improved (Table S5, Supporting Information).

## 5 Conclusion

In summary, the separation of C_3_H_6_/C_3_H_8_ is achieved via *π*‐acids of the Ag^+^ in Ag_3_pz_3_ facilitated C_3_H_6_ transport based on reversible acid–base complexation. The long‐term stability of Ag^+^ in C_3_H_6_/C_3_H_8_ separation process can be effectively overcome by utilizing the Ag_3_pz_3_ complex. In addition, this present Ag_3_pz_3_‐based PIM‐1 facilitated transport membrane appears to have good interfacial compatibility with the polymer matrix, by introducing *n*‐butyl group. A suitable organic ligand is selected to regulate the *π*‐acidity of the silver metal ions in the complex to enhance the adsorption energy between C_3_H_6_ and Ag_3_pz_3_ complex.

FIB‐SEM tomography— Thermo Fisher (FEI) Helios G4, WD 4 mm, Helios SEM immersion mode, and TLD detector were used for the FIB‐SEM experiments. The FIB, which was running at 30 kV and 900 pA, ground off sections with a nominal thickness of 50 nm. Continuous cross‐sections exposed during milling were captured by using a 30 kV secondary electron detector and magnifications of 1200–50 000×, yielding between 80 and 160 individual SEM images. The image stack was aligned with the external features of the membrane surface using a correlation algorithm, and a *y*‐direction stretching operation was performed to compensate for perspective shortening caused by the tilt angle between the sample cross‐section and the SEM detector. The different phases (i.e., PIM‐1 or Ag_3_pz_3_) were segmented in Avizo to quantify the parameters of interest from the reconstructed FIB‐SEM tomograms (FEI Visualization Sciences Group). The Supporting Information contains more details on the experimental approach.

## Conflict of Interest

The authors declare no conflict of interest.

## Supporting information

Supporting Information

Supporting Information

## Data Availability

The data that support the findings of this study are available from the corresponding author upon reasonable request.
