# Supplementary material for: Rational Design of Mixed Matrix Membranes Modulated by Trisilver Complex for Efficient Propylene/Propane Separation
Source: Adv Sci (Weinh). 2023 Feb 7;10(10):2206858. doi: 10.1002/advs.202206858 (PMC10074071; doi:10.1002/advs.202206858)
Supplement: Supplementary file 1 — Supporting Information [file ADVS-10-2206858-s002.pdf]

Supporting Information

Rational Design of Mixed Matrix Membranes Modulated by Trisilver Complex for Effcient Propylene/Propane Separation

Shenzhen Cong†, Xiaoquan Feng†, Lili Guo, Donglai Peng, Jing Wang, Jinghuo Chen, Yatao Zhang, Xiangjian Shen*, Guang Yang**

S. Cong, X. Feng, Dr. J. Wang, Prof. Y. Zhang, Dr. X. Shen
School of Chemical Engineering, Zhengzhou University, Zhengzhou 450001 (China)
E-mail: zhangyatao@zzu.edu.cn; xjshen85@zzu.edu.cn
L. Guo, Dr. J. Chen, Prof. G. Yang
College of Chemistry, Zhengzhou University, Zhengzhou 450001 (China)
E-mail: yang@zzu.edu.cn
D. Peng
School of Material & Chemical Engineering, Zhengzhou University of Light Industry, Zhengzhou 450001 (China)

† These authors contributed equally.

1. Materials and instruments

1.1 Materials

Sodium chloride (NaCl, >99.5%), sodium carbonate (Na_2CO_3, >99.8%), sodium bicarbonate ($NaHCO_3$, >99.8%), toluene (99.5%), chloroform (99%), tetrahydrofuran (THF) and ethyl acetate (99.5%) were obtained from Aladdin. H_2SO_4 (95%-98%), silver nitrate ($AgNO_3$, >99.8%), *N*,*N*-dimethylformamide (DMF, >99.5%) and methanol (MeOH, >99.5%) were purchased from Sinopharm Chemical Reagent Co., China. Anhydrous dimethylacetamide (DMAc) was obtained from J&K Chemical. Anhydrous potassium carbonate (K_2CO_3, >99%) and 4-methylpyrazole (98%) were purchased from Alfa Aesar. The above chemicals were used as received. 5, 5′, 6, 6′-Tetrahydroxy-3, 3, 3′, 3′-tetramethyl-1, 1′-spirobisindane (TTSBI, >96%, Alfa) was recrystallized by adding methylene chloride to TTSBI. 2, 3, 5, 6-Tetrafluoroterephthalonitrile (TFTPN, 99%, TCI) was dried at 150 °C under low pressure.

1.2 Instrumentation

The SEM morphology of resulting membranes were examined using Zeiss/Auriga FIB-SEM. The D8 ADVANCE X-ray diffractometer was used for X-ray diffraction (XRD) measurements at 40 kV and 40 mA for Cu Kα (λ = 1.5406) using wide-angle diffraction patterns at a scan speed of 2°/min from 5° to 60°. An AXIS Supra System X-ray photoelectron spectrometer was used to perform X-ray photoelectron spectroscopy (XPS). Al Kα is used as the excitation source, the operating voltage is 30 V, and the vacuum pressure is 10-6 Pa. Images of transmission electron microscopy (TEM) were captured using a TSM-7500F at 200 kV. True density was obtained by Micromeritics AccuPyc 1330. Ultrathin sections are obtained via cryoultramicrotome, and the cryoultramicrotome model plate is EMVC7FC7 (Leica). N_2, C_3H_6 and C_3H_8 adsorption tests were investigated by using an BSD-PM2. The C_3H_6 and C_3H_8 adsorption tests were evaluated at 298 K. The tested membrane (about 200

mg) to be tested was cut into small pieces, and then degassed for 12 h under high vacuum degree (< 10^{-6} bar) at 120 °C prior to the examination.

FIB-SEM tomography:

Experiments in FIB-SEM were carried out using a Zeiss AURIGA Compact microscope. The membranes were first sputter coated with a ca. 4 nm Au layer. The Ga^+ FIB, working at 30 kV and 900 pA, was used to create a ca. 20 µm-wide trench on the top surface of the membranes. An automated routine programmed in Run Script software with drift correction was used to perform serial sectioning and SEM imaging. The FIB milled away slices with nominal thicknesses of 50 nm while operating at 30 kV and 900 pA. At magnifications ranging from 1200 to 50000, individual SEM micrographs of the consecutive cross-sections exposed during milling were captured using a 30 kV Secondary Electron Detector in the SEM.

Dataset processing and tomogram reconstruction

Reconstruct the 3D internal structure of the membrane using a collection of serial SEM micrographs. Using a cross-correlation algorithm, align the image stack with the external features of the membrane surface. For further analysis, select and crop the central region of the FIB exposed face of the trench. Then, stretch in the y-direction to compensate for the foreshortening caused by the tilt angle between the sample cross-section and the SEM detector. A bandpass filter was applied to the resulting aligned stack to reduce shadowing effects by the trench walls as well as vertical curtaining, followed by mean-field filtering (with a circular kernel) to enhance the contrast of the embedded Ag_3pz_3 complex relative to the polymeric matrix, and thereby facilitate subsequent image segmentation. The final 3D reconstructions displayed non-cubic voxels with *x*:*y*:*z* dimensions of (10.8-24.5):(13.8-24.5):52 nm.

X-ray Crystallography. A suitable crystal was chosen and mounted on a cryosphere with a vaseline coating. Utilizing 298 K monochromatic Mo Kα radiation from graphite (λ = 0.7107

), single crystal X-ray diffraction data were obtained on a Bruker AXS GmbH diffractometer. A multi-scan method was used to correct for absorption. The SHELXTL software package was used to solve all structures using the direct method and refine them using full matrix least squares based on F2. (*1-3*) Anisotropic displacement parameters were used to refine all non-H atoms. Geometric generation produces H-atoms.

Table S1 contains a list of the crystallographic data. CCDC 1916024 is available for the supplementary crystallographic data of $Ag_3(BuOOCdnpz)_3$. The Cambridge Crystal Data Centre is offering these data free of charge at www.ccdc.cam.ac.uk/data_request/cif.

2. Fabrication of the facilitated transport membrane

2.1 Synthesis of PIM-1

PIM-1 was synthesized under circumstances resembling the method described by Du et al. (*4*) TTSBI (3.404 g, 10 mmol), TFTPN (2.001 g, 10 mmol), anhydrous potassium carbonate (4.140 g, 30 mmol), DMAc (20 mL under nitrogen atmosphere), and toluene were put into a dry 100 mL three-neck round bottom flask with a Dean-Stark trap (ca. 6 mL). The flask was then moved into an oil bath that was heated to 160°C while being mechanically stirred. Pour the mixture into 200 mL of methanol after the polymerization is finished to get an elastic, linear, pale yellow polymer. The polymer product was dissolved in chloroform and reprecipitated in methanol. This process is carried out twice. The resulting polymer underwent a 24-hour vacuum drying process at 120 °C after being refluxed in deionized water for 4-5 hours.

2.2 Synthesis of Butyl 3, 5-dinitro-1*H*-pyrazole-4-carboxylate (BuOOCdnpzH)

Keeping the temperature below 35 °C, 3.28 g of 4-methylpyrazole was dissolved in 40 mL of concentrated sulfuric acid. At 30–40 °C, fuming nitric acid (10 mL) was vigorously stirred into the mixture. After another 8 hours of reaction time at 90 to 100 °C, the mixture was cooled and put into 250 mL of ice water. The resulting suspension was bubbled through with

air to remove nitrogen dioxide. Sodium bicarbonate was then added to the mixture to neutralize it, and ethyl acetate was then extracted from the mixture (3×20 mL). Under reduced pressure, the organic extract was distilled, and the solid residue was re-crystallized using ethanol and water. Yield: 5.50 g (80%). Mp.:180-182°C. ^{1}H NMR (400 MHz, DMSO) δ 2.57 (s, 1 H) ppm. ^{13}C NMR (101 MHz, CD_3CN) δ 163.86 (s), 154.11 (s), 118.40 (s), 107.70 (s), 66.59 (s,), 31.19 (s), 19.75 (s), 13.93 (s). FTIR (KBr pellet) ν_{max} (cm^{-1}): 3238 (*v*), 2959 (*w*), 1598 (*m*), 1547 (*s*, ν_{asNO_2}), 1489 (*m*), 1410 (*m*), 1393 (*m*), 1336 (*s*, ν_{sNO_2}), 1232 (*m*), 1174 (*v*), 1082 (*v*), 965 (*v*), 843 (*s*), 758 (*m*).

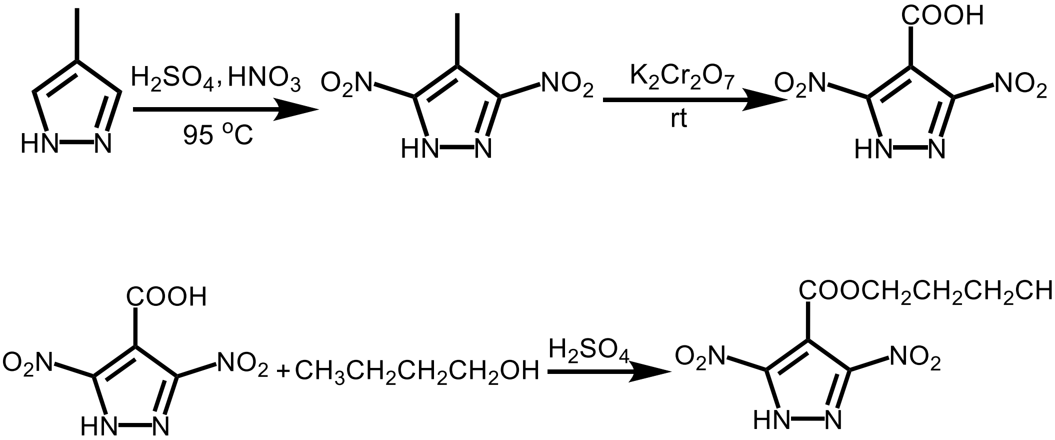

Scheme S1 Synthetic route of butyl 3, 5-dinitro-1*H*-pyrazole-4-carboxylate

2.3 Synthesis of the trinuclear complex-Ag_3(BuOOCdnpz)$_3$

Butyl 3,5-dinitro-1*H*-pyrazole-4-carboxylate (12.9 mg, 0.05 mmol) and $AgNO_3$ (8.5 mg, 0.05 mmol) were dissolved in 4 mL of methanol. The resulting solution was stirred at room temperature for 2 h, during which time a white precipitate gradually formed (Scheme S1). After filtration, the obtained microcrystals were washed twice with methanol, and then dried under vacuum. Slow evaporation of the THF solution of the white powdery sample afforded crystals of Ag_3(BuOOCdnpz)$_3$ (denoted as Ag_3pz_3) suitable for X-ray analysis. Yield: 27.4 mg, 0.025 mmol, 50%. ^{1}H NMR (400 MHz, CD_3CN) δ 4.33 (t, *J* = 6.6 Hz, 1 H), 1.69 (dt, *J* = 14.5, 6.6 Hz, 1 H), 1.49 – 1.30 (m, 1 H), 0.93 (t, *J* = 7.4 Hz, 1 H).^{13}C NMR (101 MHz, CD_3CN) δ 162.57 (s), 153.06 (s), 107.91 (s), 66.89 (s), 31.14 (s), 19.70 (s), 13.91 (s). FTIR (KBr pellet)

ν_{max} (cm^{-1}): 3437 (*m*), 2964 (*m*), 2874 (*w*), 1751 (*w*), 1701 (*s*,$\nu_{C=O}$), 1523 (*s*,ν_{NO_2}), 1430 (*m*), 1327 (*s*,ν_{NO_2}), 1274 (*m*), 1110 (*s*,$\nu_{asC\text{-}O\text{-}C}$), 920 (*m*), 861 (*s*), 564(*m*).

2.4 Preparation of facilitated transport membranes

A 0.45 μm PTFE filter was used to filter a 4 wt% PIM-1 solution in tetrahydrofuran before casting it onto a horizontal glass plate to create a PIM-1 polymer membrane that isn't filled with any material. Slowly evaporating the solvent at room temperature led to the creation of a dense membrane. The remaining solvent was then removed from the membrane by placing it in a vacuum oven for 24 hours at 120 °C. To prepare the facilitated transport membrane with nanocrystals of Ag_3pz_3 as filler, THF was used as a solvent to dissolve Ag_3pz_3. Subsequently, the THF solution of Ag_3pz_3 was added to a THF solution of PIM-1. Prior to casting, the mixture was stirred for 12 hours and sonicated for roughly 10 minutes. The membrane was initially evaporated, and any remaining solvent was then removed by placing it in a vacuum oven at 120 °C for 24 hours. The membranes were produced with a thickness between 70 and 90 μm. The membranes are identified as Ag_3pz_3/PIM-1-X, where X denotes the Ag_3pz_3 mass percentage.

3. Computational Methods

All ab initio total energy calculations for describing the simulated interaction between C_3H_6 (C_3H_8) and $Ag_3(BuOOCdnpz)_3$ were made using density functional theory (DFT) within the framework of the VASP (Vienna ab initio Simulation Packages) code, (*5*, *6*) which uses a plane wave basis set for the electronic orbitals. Using the Perdew-Burke-Ernzerhof (PBE) function, the generalized gradient approximation describes the electronic exchange and correlation. *(7)* The projector augmented-wave (PAW) method was used to study how valence electrons interact with ionic cores. (*8*) With a cubic box of 30.0 Å × 30.0 Å × 25.0 Å, a sizable supercell was modeled. K-points 3 × 3 × 1 used a Monkhorst-Pack grid. (*9*) A cut-off energy of 450 eV was employed. A 450 eV cut-off energy was used.The Methfessel-Paxton

scheme was extended to include electronic smearing with N = 0 and = 0.2 eV. (*10*) To take the dispersion forces into account, we have considered the van der Waals correlation function in this work. (*11*) The adsorption energy of C_3H_6 is defined as E_{ads} = E(model+C_3H_6)-E(model)-E(C_3H_6), where E(model+C_3H_6) and E(C_3H_6) are the total energies of the system of C_3H_6 adsorption on the Ag_3(BuOOCdnpz)$_3$ model, the system of Ag_3(BuOOCdnpz)$_3$ model and the C_3H_6 molecule, respectively.

Molecular Dynamics (MD) Simulations. In this work, CHARMM36m force field (*12*) and GROMACS software (*13*) (version 2019.6) were used for all all-atom MD simulations to capture the diffusion dynamics of C_3H_6 and C_3H_8 through PIM-1 (n=4) and Ag_3pz_3/PIM-1 porous membranes. Parameterizations of C_3H_6, C_3H_8, PIM-1 (n=4) and Ag_3pz_3 molecules were done based on CGenFF (*14*) and Interface force field (*15*). PIM-1 and Ag_3pz_3/PIM-1 porous membranes were set up by periodically placing PIM-1 and Ag_3pz_3 molecules. For Ag_3pz_3/PIM-1 porous membranes, the ratio of Ag_3pz_3:PIM-1 is 1:3, which makes Ag_3pz_3 account for ~10wt% of the whole membrane. In order to study the diffusion dynamics of C_3H_6 and C_3H_8, a 6nm×6nm×6nm C_3H_6/C_3H_8 pool was placed at the entrance of PIM-1 and Ag_3pz_3/PIM-1 porous membranes (6nm×6nm×16nm). Subsequently, 10ns all-atom MD simulation with the time step of 2fs was performed for each system to capture the free diffusion process of C_3H_6 and C_3H_8 through the porous membranes.

4. Gas permeation tests

A facilitated transport mechanism can account for the gas permeation characteristics of dense membranes. The constant volume method is used to calculate the pure gas permeability. (*16*) Gases were tested in the order of C_3H_6 and C_3H_8 at 1 atm and 30°C. Equation 1 is used to calculate the gas permeability (P) from the steady rate of pressure increase downstream ($^{d_p}/_{d_t}$):

$$P = \frac{273\times10^{10}}{760}\frac{Vl}{AT(P_2\times\frac{76}{14.7})}\frac{dp}{dt} \qquad (1)$$

where V is the downstream cavity's volume (cm^3) and l is the thickness of membrane (cm). A stands for the effective test area (cm^2) of the membrane, T for the operational temperature (K), and P_2 for the upstream operational pressure (psi). The gas permeate test chamber is always maintained in a vacuum state and it can be approximated that there is no permeate gas in this section of the equipment. Therefore, formula (1) can be used to calculate the gas permeability. In addition, the rate of facilitated transport is much greater than passive sorption and diffusion, and the passive transport process can be ignored.

The mixed gas separation performance was evaluated by a Wicke–Kallenbach technique by using a binary gas mixture feed (50/50 C_3H_6/C_3H_8). To examine the permeate side, a gas chromatograph (Shimadzu GC-2014C) was employed. The membrane was put to the test with Ar serving as the sweep gas at 303 K and a total gas feed pressure of 1 bar. The permeability of component i was calculated as:

$$P_{\mathrm{i}} = \frac{Q_i l}{\Delta P_i A} \qquad (2)$$

Q_i is volume flow rate under standard conditions, the membrane area is A, its thickness is l, and the partial pressure difference of component i across the membrane is Δp_i.

The separation factor α was calculated as:

$$\alpha_{C_3H_6/C_3H_8} = \frac{P_{C_3H_6}}{P_{C_3H_8}} \qquad (3)$$

Table S1 Crystal data and structure refinement for as-synthesized $Ag_3(BuOOCdnpz)_3$.

	$Ag_3(BuOOCdnpz)_3$
Formula	$C_{24}H_{27}Ag_3N_{12}O_{18}$
Mol. Wt.	1095.18
T [K]	298
Crystal system	triclinic
Space group	P-1
a [Å]	10.20 (2)
b [Å]	13.04 (2)
c [Å]	14.75 (2)
α [°]	105.86 (5)
β [°]	97.44 (4)
γ [°]	97.02 (5)
V [Å^3]	1846 (5)
Z	2
$\rho_{calcd.}$ [mg m^{-3}]	1.971
μ [mm^{-1}]	1.668
F(000)	1080.0
Crystal size / mm^3	0.25 × 0.22 × 0.21
Radiation	MoKα (λ = 0.71073)
Reflection collected	19003
Unique reflections	6479 (R_{int} = 0.0417)
Final R indices [$I > 2\sigma(I)$]	R_1 = 0.0465, wR_2 = 0.1177
Final R indices [all data]	R_1 = 0.0784, wR_2 = 0.1373

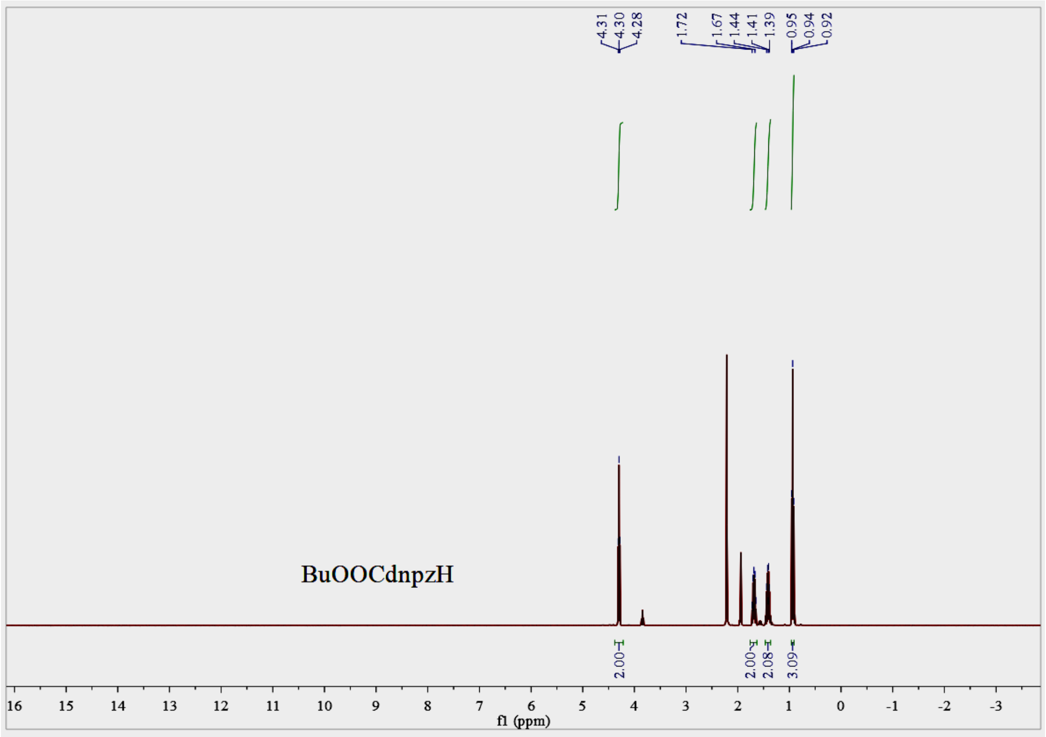

Figure S1 ^{1}H NMR spectrum of the organic ligand BuOOCdnpzH in CD_3CN at room temperature.

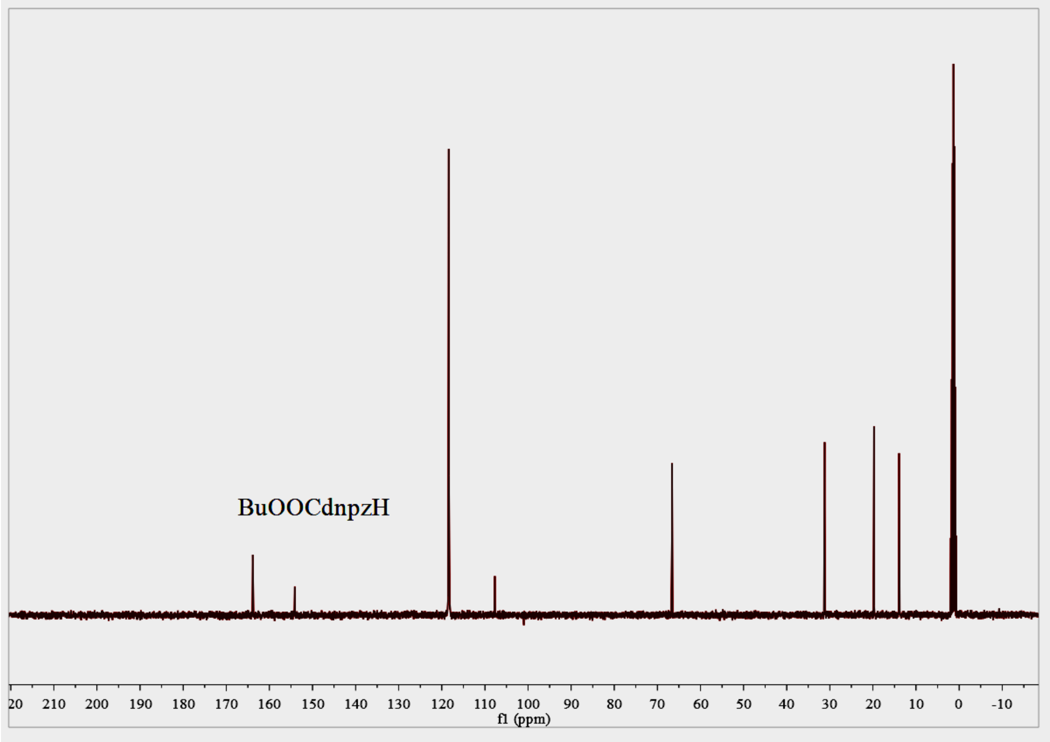

Figure S2 ^{13}C NMR spectrum of the organic ligand BuOOCdnpzH in CD_3CN at room temperature.

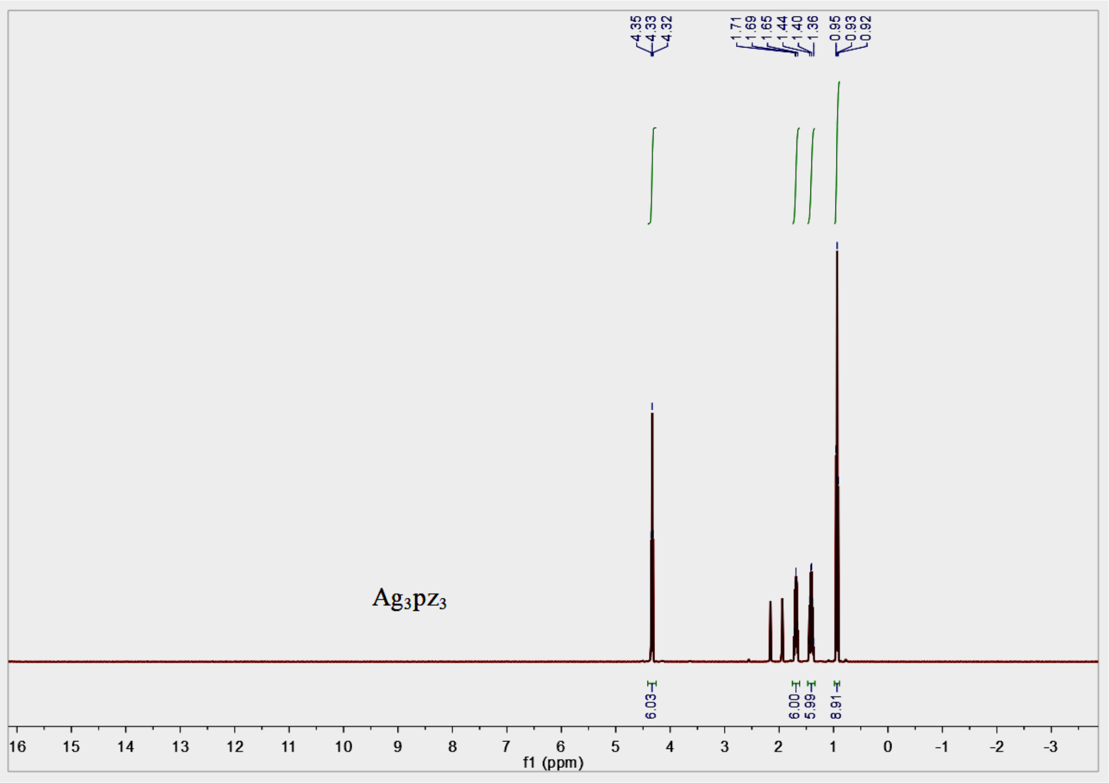

Figure S3 ^{1}H NMR spectrum of Ag_3pz_3 in CD_3CN at room temperature.

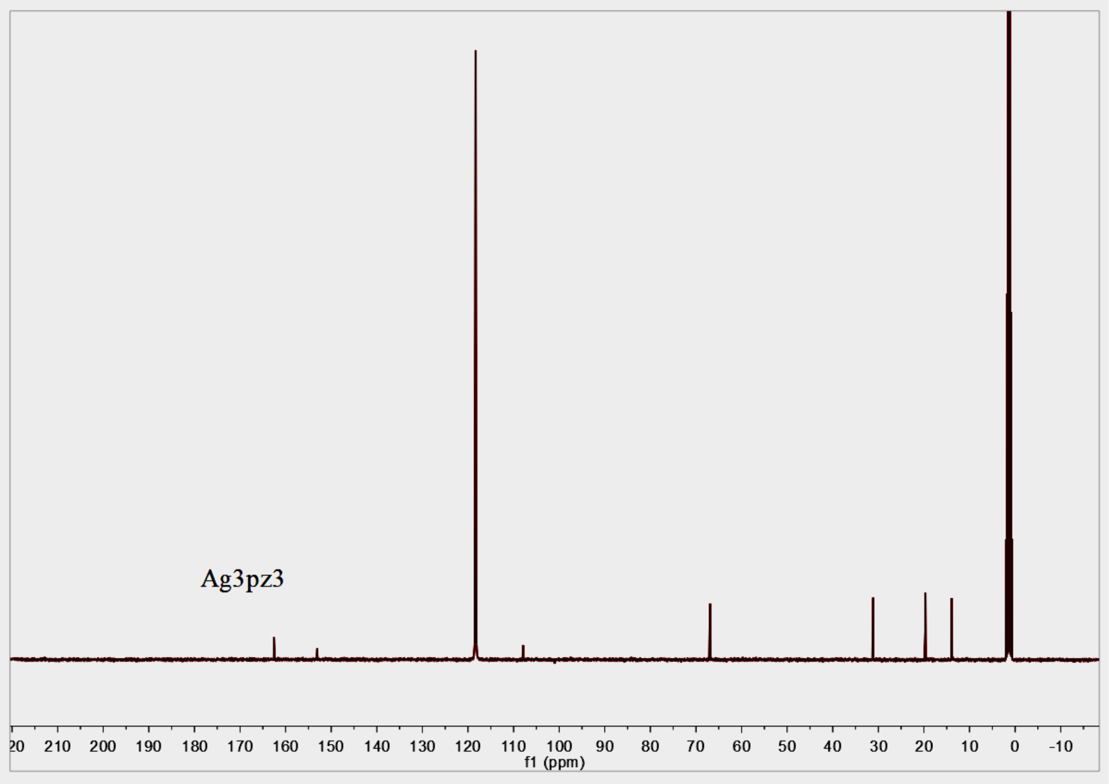

Figure S4 ^{13}C NMR spectrum of Ag_3pz_3 in CD_3CN at room temperature.

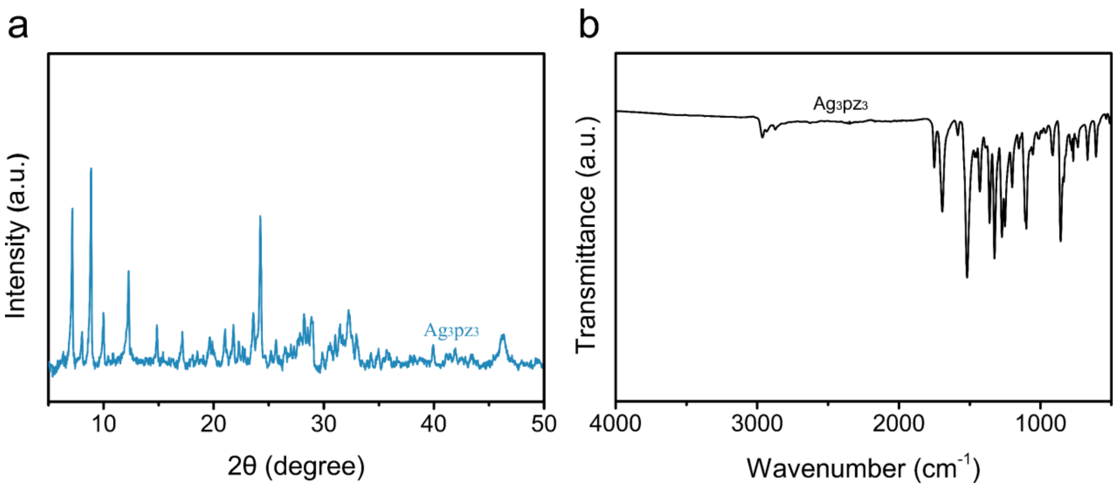

Figure S5 (a) XRD patterns of the Ag_3pz_3 complex; (b) FTIR spectra of the Ag_3pz_3 complex.

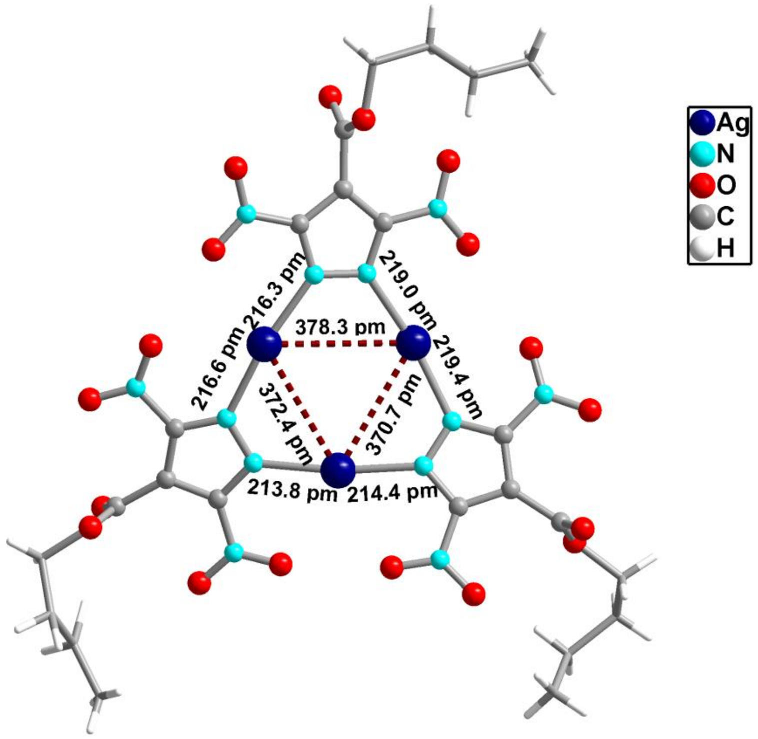

Figure S6 Molecular structure of Ag_3pz_3 determined by single crystal X-ray diffraction.

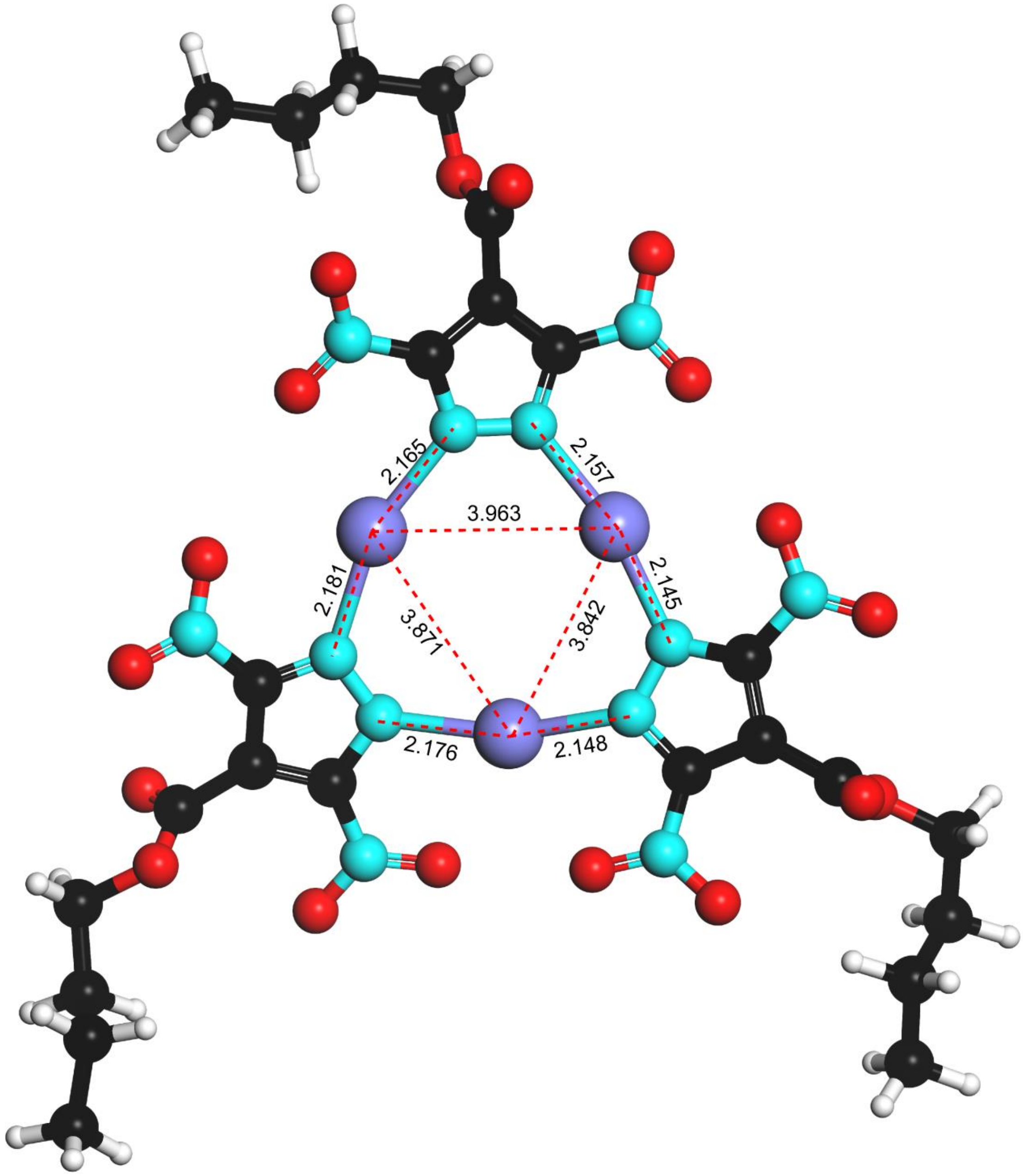

Figure S7 Molecular structure of Ag_3pz_3 based on DFT calculations. The distance between the Ag_3pz_3 central ion and coordinating atom. The distance between the Ag_3pz_3 center ions. (The unit of distance is Angstrom)

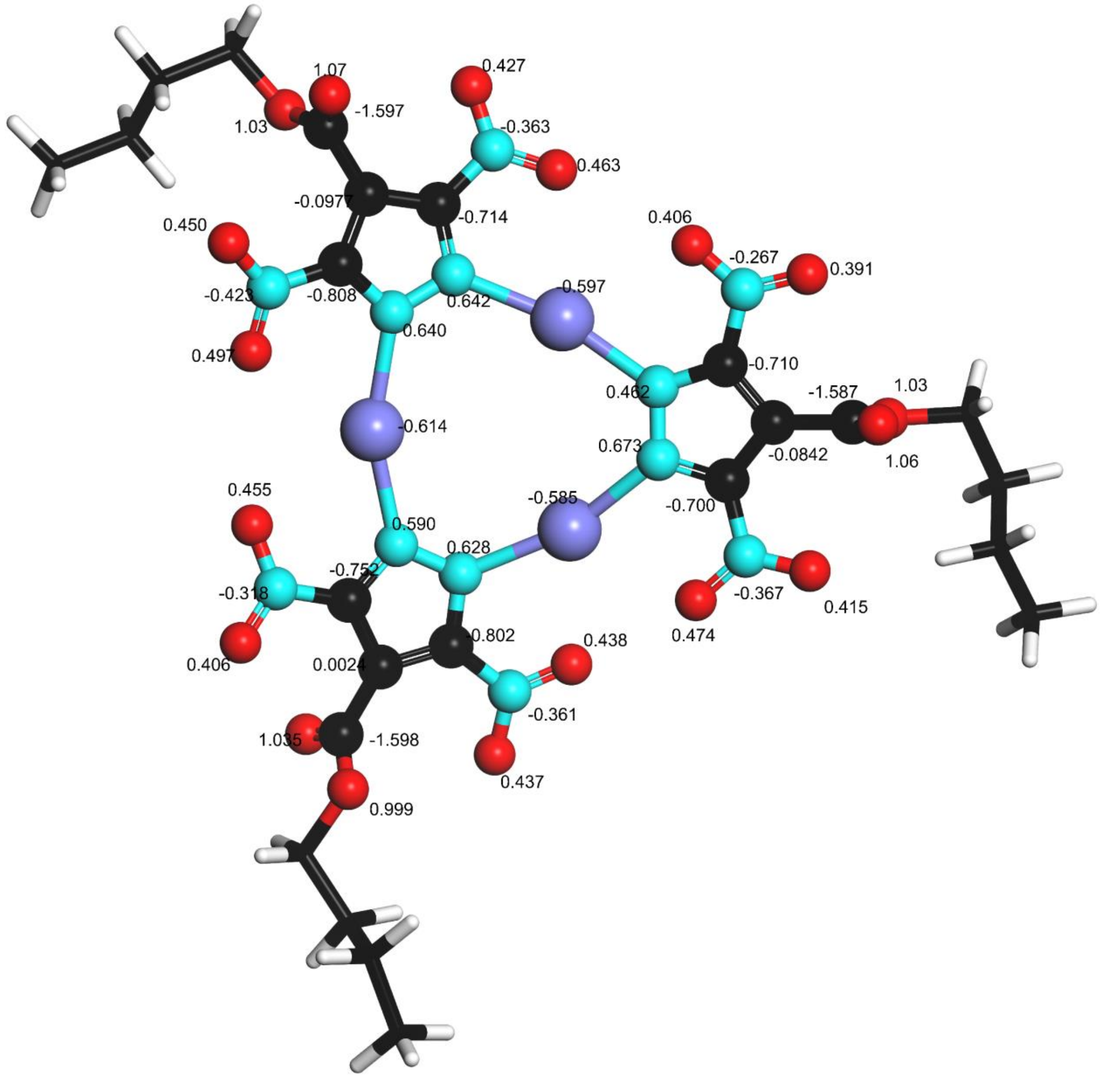

Figure S8 Bader charge analysis results for Ag_3pz_3.

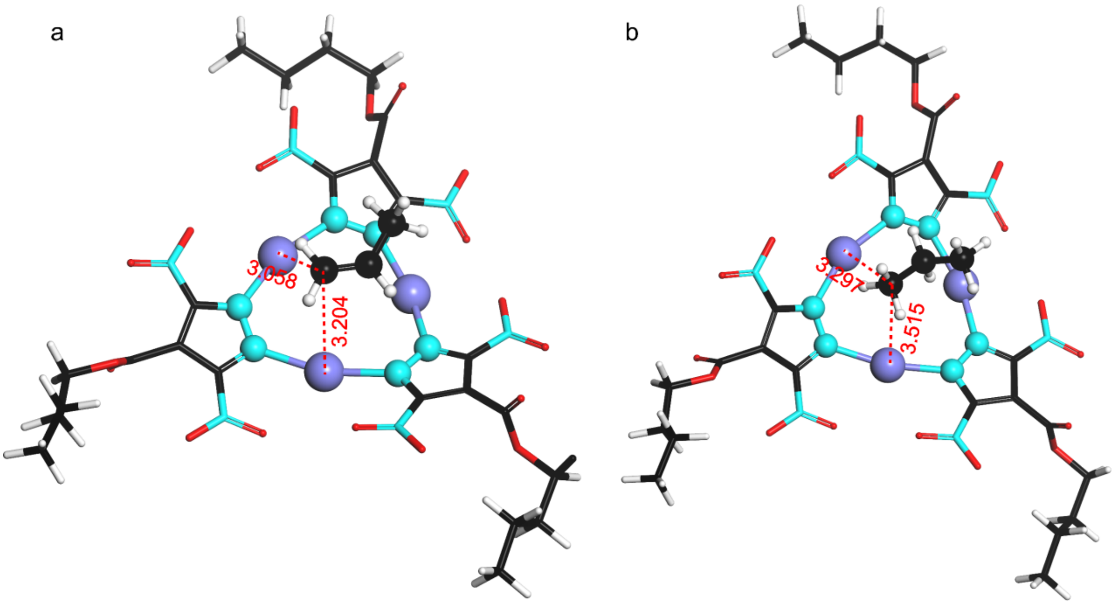

Figure S9 Schematic picture showing optimized C_3H_6 and C_3H_8 adsorption on the Ag_3pz_3 model: (a) The process of facilitated transport of Ag_3pz_3 for C_3H_6. The distances of C_3H_6 and two Ag^+ during the transport process with an adsorption energy of -30.2 kJ/mol; (b) The process of facilitated transport of Ag_3pz_3 for C_3H_8. The distances of C_3H_8 and two Ag^+ during the transport process with an adsorption energy of -25.6 kJ/mol.

Table S2 The adsorption energies (in eV) of C_3H_6 and C_3H_8 adsorption on different positions of Ag_3pz_3 complex.

	Top of the single Ag^+	Center of the triangle Ag^+
C_3H_6	-0.400	-0.314
C_3H_8	-0.046	-0.266

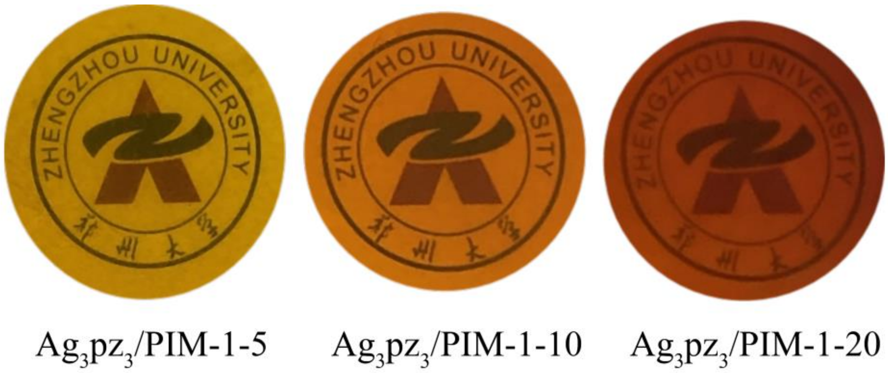

Figure S10 Optical images of Ag_3pz_3/PIM-1 membranes. The membrane's color progressively deepens when Ag_3pz_3 loading is increased.

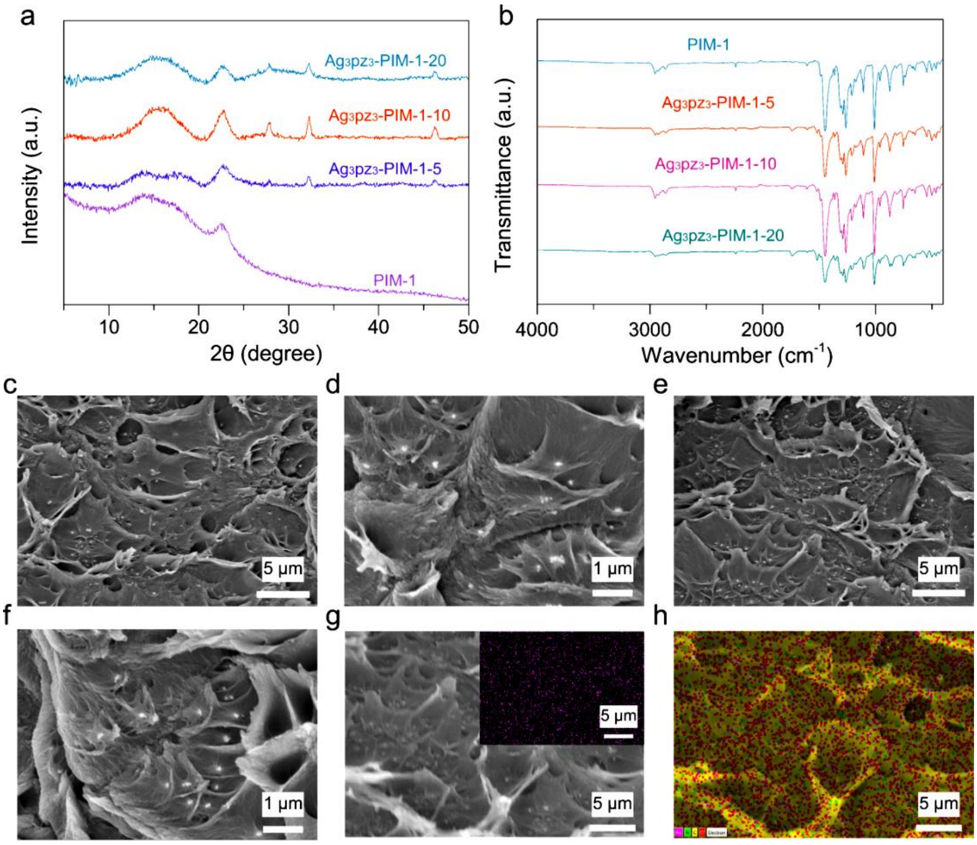

Figure S11 Characterization of Ag_3pz_3 complex and facilitated transport membranes: (a) XRD patterns of pristine PIM-1 membrane and Ag_3pz_3/PIM-1 facilitated transport membrane with 0 wt%, 5 wt%, 10 wt%, 20 wt% Ag_3pz_3 loading; (b) FTIR spectra of PIM-1 and Ag_3pz_3/PIM-1 with 5 wt%, 10 wt%, 20 wt% Ag_3pz_3 loading; (c-h) SEM images of the Ag_3pz_3/PIM-1 facilitated transport membranes; (c, d) Facilitated transport membrane with Ag_3pz_3 loading of 5 wt%; (e, f) Facilitated transport membrane with Ag_3pz_3 loading of 20 wt%; (g, h) SEM-EDS of Ag_3pz_3/PIM-1 with 10 wt% loading (pink, Ag element).

The XRD results of PIM-1 membrane indicated two polymer peaks corresponding to *d*-spacing of 6.5 Å and 4.0 Å, which were attributed to micropores between polymer chains and efficiently packed chains, respectively. The XRD analysis of obtained Ag_3pz_3/PIM-1 membranes show that the characteristic peak of Ag_3pz_3 could not be distinguished. Here, the Ag_3pz_3 is buried within polymer segments, and the quantum size effect of Ag_3pz_3 also affects the crystal diffraction of Ag_3pz_3. In addition, the *d*-spacing of the polymer segment increases with incorporated Ag_3pz_3. Analysis of the FTIR of the obtained Ag_3pz_3/PIM-1 membranes show characteristic C=O and $-NO_2$ absorptions, indicating that Ag_3pz_3 is incorporated. The SEM-EDS of a Ag_3pz_3/PIM-1-10 membrane cross-section was measured, which shows that Ag element is homogenously distributed within the membrane.

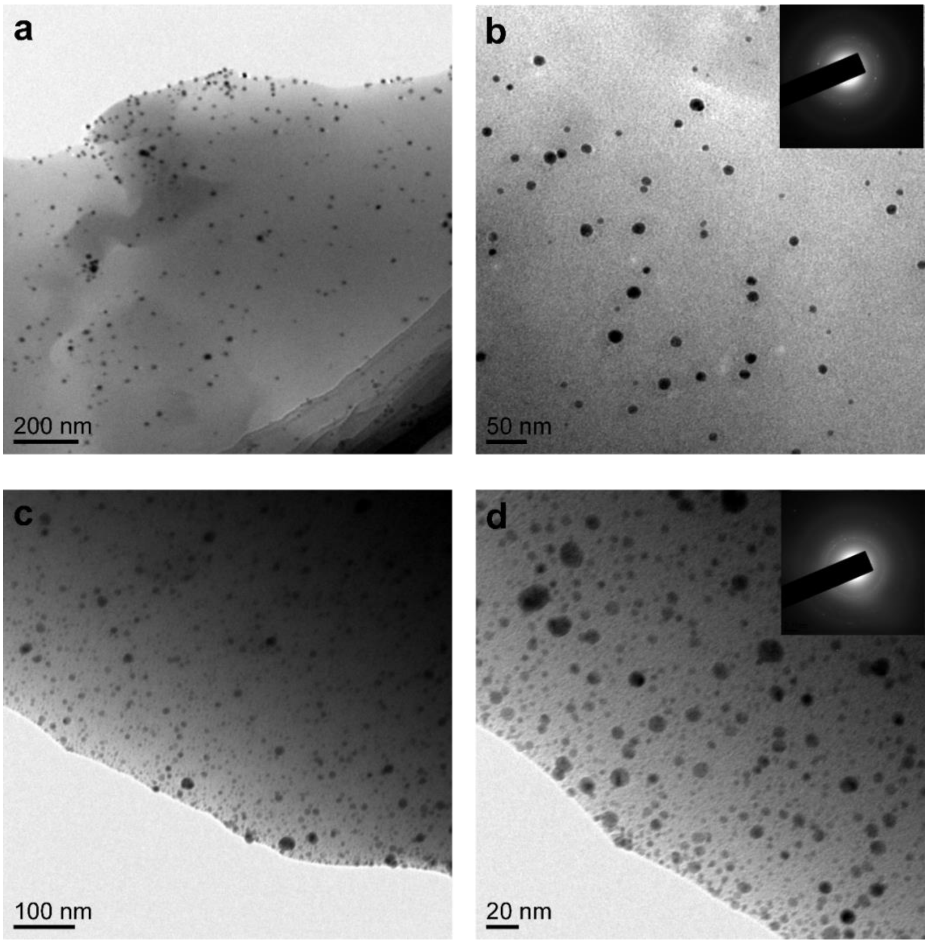

Figure S12 (a, b) Cross-sectional TEM images of Ag_3pz_3/PIM-1-10 membrane; (c, d) Cross-sectional TEM images of an Ag_3pz_3/PIM-1-20 ultrathin section (inset: the selected area electron diffraction (SAED) of Ag_3pz_3/PIM-1 membrane indicates that the crystal structure of Ag_3pz_3 retains integrity).

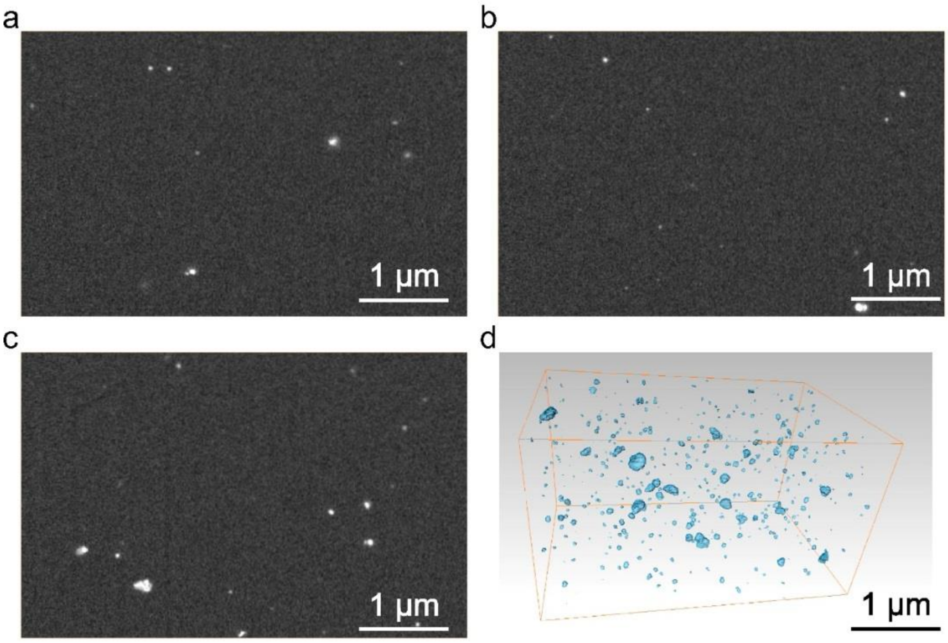

Figure S13 Representative cross-sectional SEM micrographs of composite membranes containing Ag_3pz_3 embedded in PIM-1 (On the dark gray polymer matrix, Ag_3pz_3 particles appear as bright spots): (a) 80th piece, (b) 100th piece, (c) 120th piece. Ag_3pz_3 complexes are clearly seen in the SEM micrographs of cross-sections; (d) A surface-rendered view of a segmented FIB-SEM tomogram of an Ag_3pz_3-embedded composite membrane in PIM-1. (The box in (d) has dimensions of 5.1:3.2:2.8 μm in the x:y:z directions and is depicted with blue Ag_3pz_3 particles).

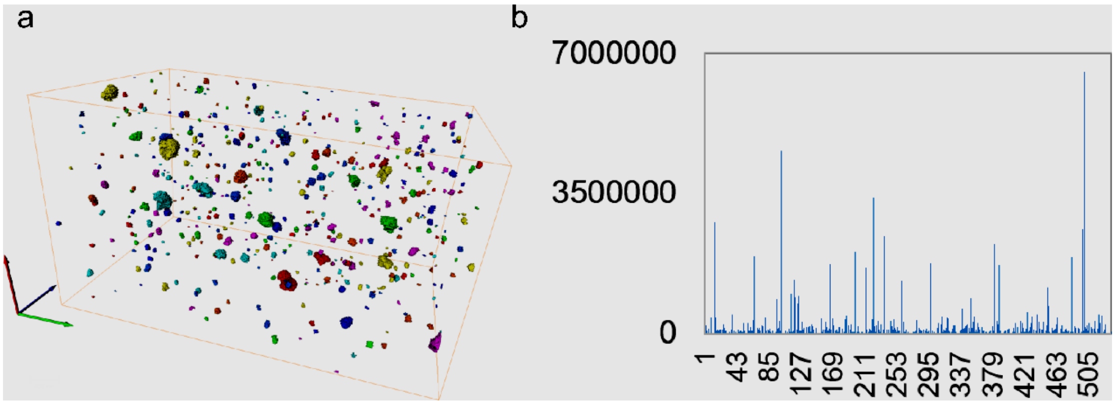

Figure S14 (a) A 3D reconstruction was used to create an orthogonal cross-section of the FIB-SEM tomogram of the Ag3pz3/PIM-1-10 membrane. (b) Size distribution of Ag_3pz_3 complex. The abscissa indicates the volume size (nm^3) and the ordinate is the total volume of the particle size with the volume of the abscissa.

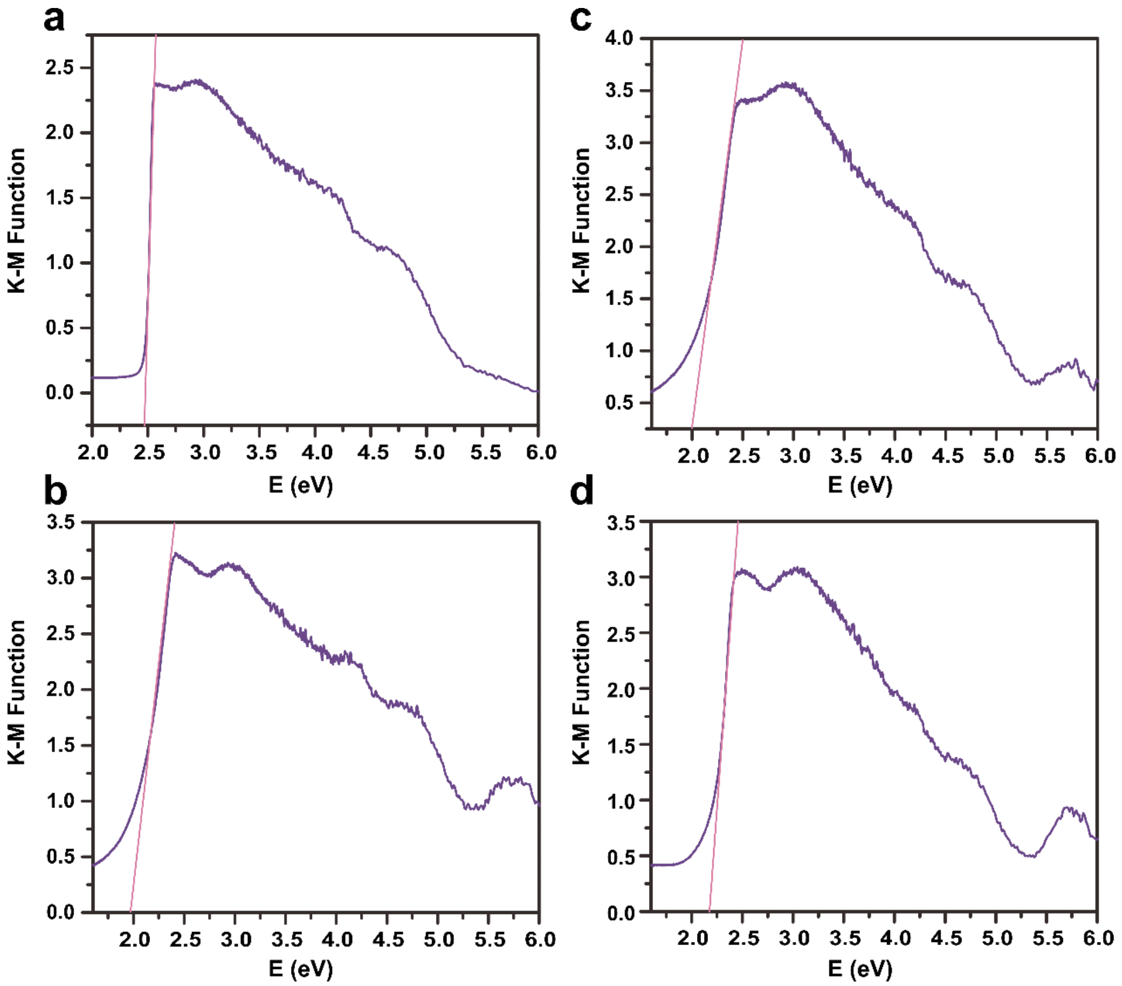

Figure S15 The curves of K-M Function vs E for Ag_3pz_3/PIM-1 membrane: (a) The pristine PIM-1 membrane; (b) The Ag_3pz_3/PIM-1-5 membrane; (c) The Ag_3pz_3/PIM-1-10 membrane; (d) The Ag_3pz_3/PIM-1-20 membrane.

Table S4 The major UV-Vis bands of the Ag_3pz_3/PIM-1 membrane.

Sample	Wavelength/nm
Ag_3pz_3/PIM-1-5	213~215
Ag_3pz_3/PIM-1-10	213~215, 217~218
Ag_3pz_3/PIM-1-20	215~217, 219~220

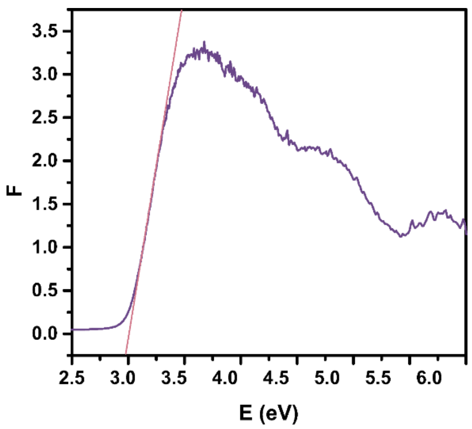

Figure S16 The curves of K-M Function vs. E for Ag_3pz_3 powder.

The curves of K-M Function (based-on UV-Vis adsorption) of PIM-1 indicated that the bandgap width is 2.42 eV. However, the Ag_3pz_3 filler material is incorporated into the polymer matrix, and the conduction band of the Ag_3pz_3/PIM-1 membrane is raised, which indicates that the membrane material exhibits properties of a semiconductor.

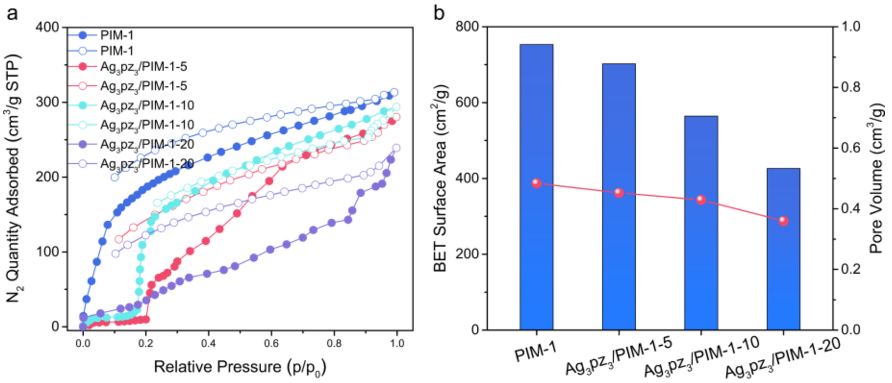

Figure S17 (a) N_2 adsorption-desorption isotherms for Ag_3pz_3/PIM-1-x MMMs; (b) structural properties of Ag_3pz_3/PIM-1-x MMMs.

The N_2 (77.3 K) adsorption-desorption isotherms can be used to qualitatively evaluate how the Ag_3pz_3 packing affects the microstructure of the manufactured MMMs. From N_2 adsorption-desorption isotherms, total pore volume and BET surface area were calculated (Figure S17). The pore volume and surface area tend to decrease with the Ag_3pz_3 loading. The n-butyl group of Ag_3pz_3 can fill the pores and reduce the polymer, the surface area and pore volume of the Ag_3pz_3/PIM-1-10 MMMs were reduced by 25.1% and 11.4%, respectively, in comparison to the pure PIM-1 membrane. The FFV of the resulting MMMs is further decreased by the porosity and chain spacing.

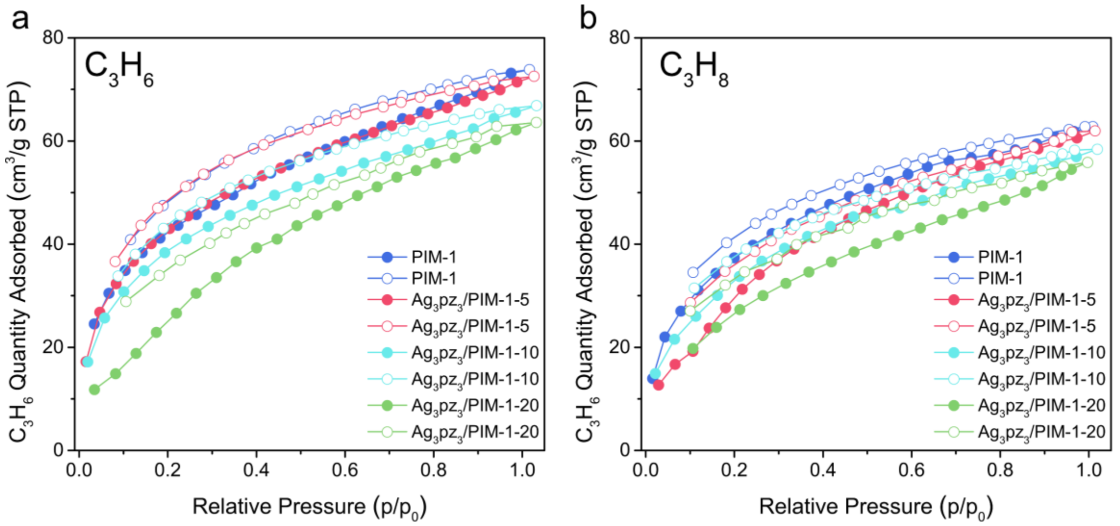

Figure S18 The PIM-1 membrane and Ag_3pz_3/PIM-1-x MMMs C_3H_6 (a) and C_3H_8 (b) adsorption/desorption isotherms were evaluated at 298 K. (Solid symbol is adsorption and hollow symbol is desorption.)

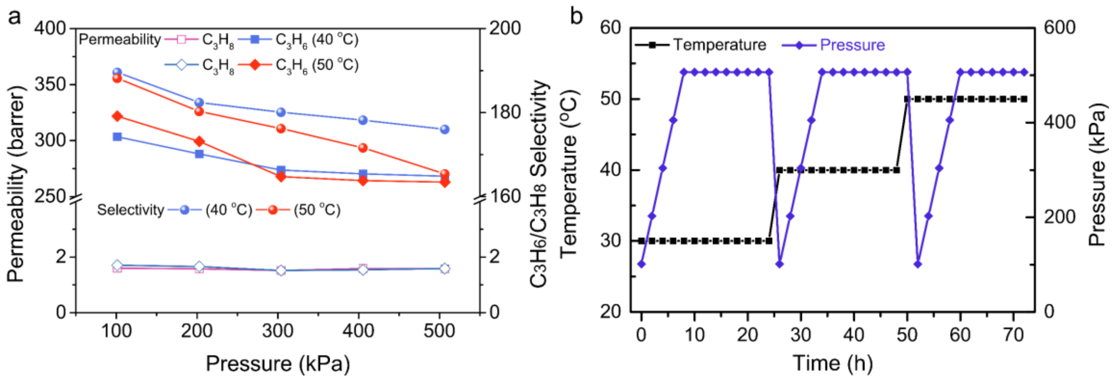

Figure S19 (a) C_3H_6 and C_3H_8 permeability and C_3H_6/C_3H_8 selectivity of Ag_3pz_3/PIM-1-10 membrane at different temperatures and pressure; (b) test conditions and testing period of the Ag_3pz_3/PIM-1-10 membrane.

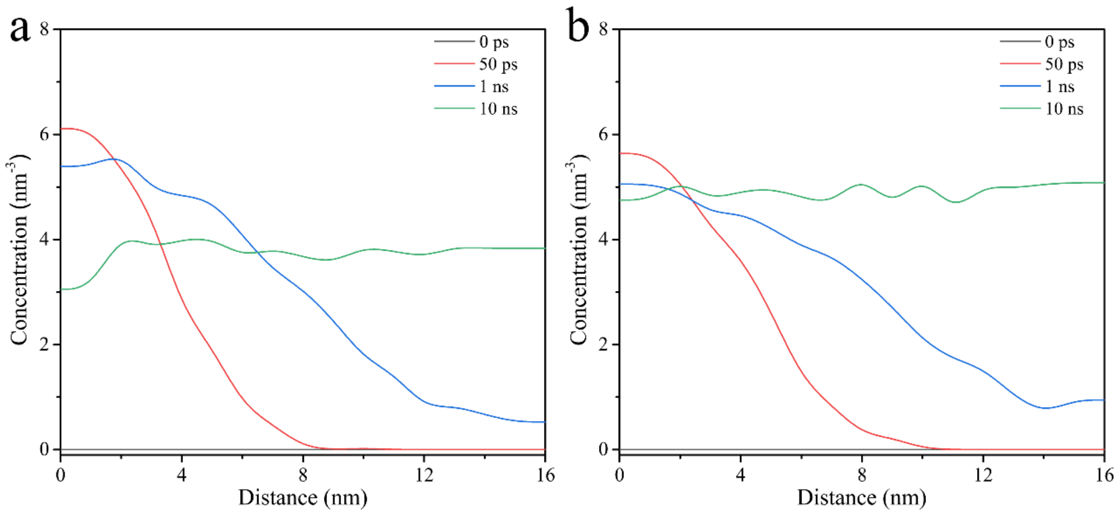

Figure S20 Concentration distribution diagram of C_3H_6 (a) and C_3H_8 (b) in PIM-1 at different times.

To explore whether the triangular Ag_3pz_3 complex can survive during the preparation of PIM-1 membrane and the separation of propylene/propane, the original PIM-1 membrane, Ag_3pz_3/PIM-1-20 membrane and Ag_3pz_3/PIM-1-20 membrane by the propylene test were immersed in acetonitrile for 12 h. The acetonitrile solutions were separated and placed at ambient temperature for 12 h to afford colorless crystals, which were then analyzed by ^{1}H NMR spectroscopy. Figure S17 and S18 show that the NMR spectra of these crystals are the same as that of the original Ag_3pz_3 complex, implying that Ag_3pz_3 retains its structural integrity during the treatments described in above. ^{1}H NMR (400 MHz, CD_3CN) δ 4.33 (t, J = 6.6 Hz, 1 H), 1.69 (dt, J = 14.5, 6.6 Hz, 1 H), 1.49 – 1.30 (m, 1 H), 0.93 (t, J = 7.4 Hz, 1 H). The Ag_3pz_3 powder was obtained via acetonitrile-soaking from Ag_3pz_3/PIM-1-20 membrane already used for the propylene test. ^{1}H NMR (400 MHz, CD_3CN) δ 4.33 (t, J = 6.6 Hz, 1 H), 1.69 (dt, J = 14.5, 6.6 Hz, 1 H), 1.48 – 1.35 (m, 1 H), 0.94 (t, J= 7.4 Hz, 1 H).

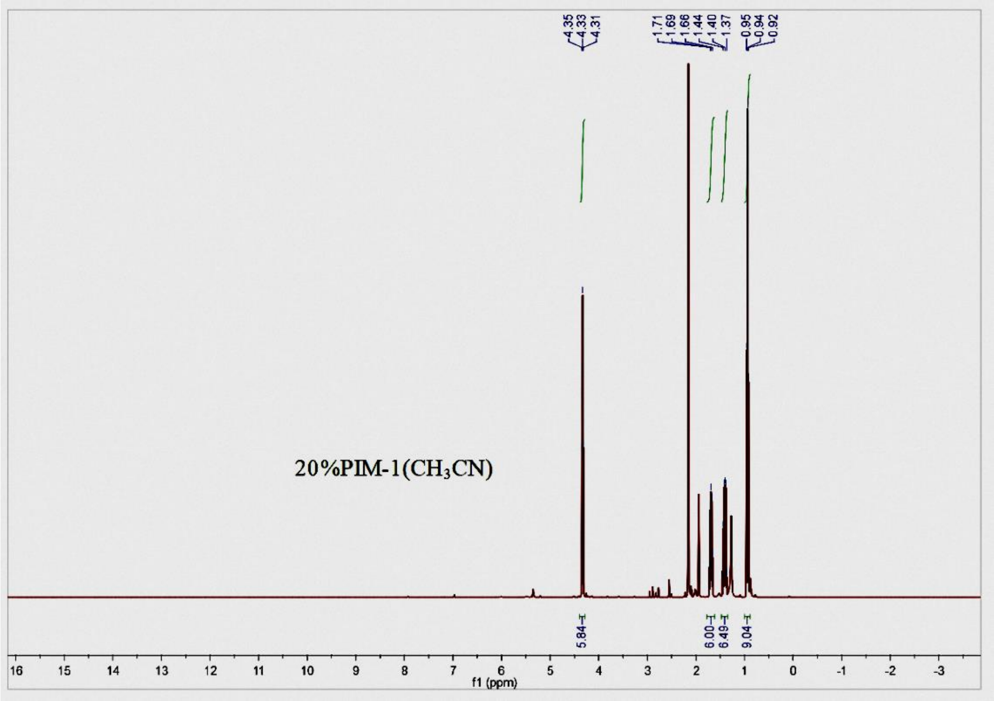

Figure S21 ^{1}H NMR spectrum of the sample released by acetonitrile-soaking from Ag_3pz_3/PIM-1-20 membrane. (in CD_3CN at room temperature)

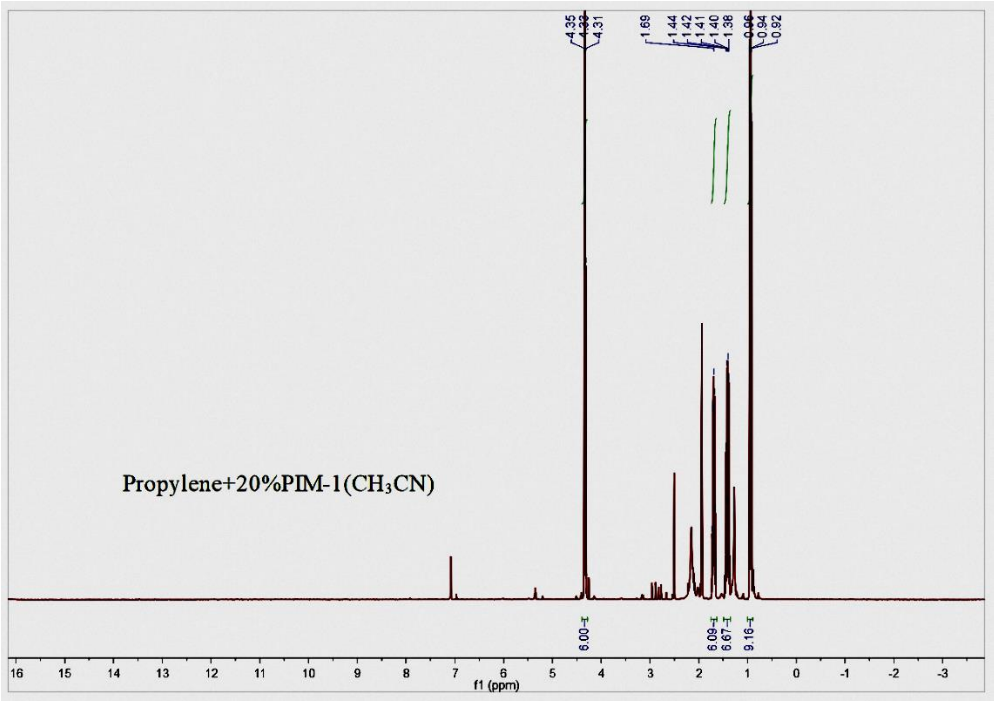

Figure S22 ^{1}H NMR spectrum of the sample released by acetonitrile-soaking from Ag_3pz_3/PIM-1-20 membrane already used for the propylene test. (in CD_3CN at room temperature)

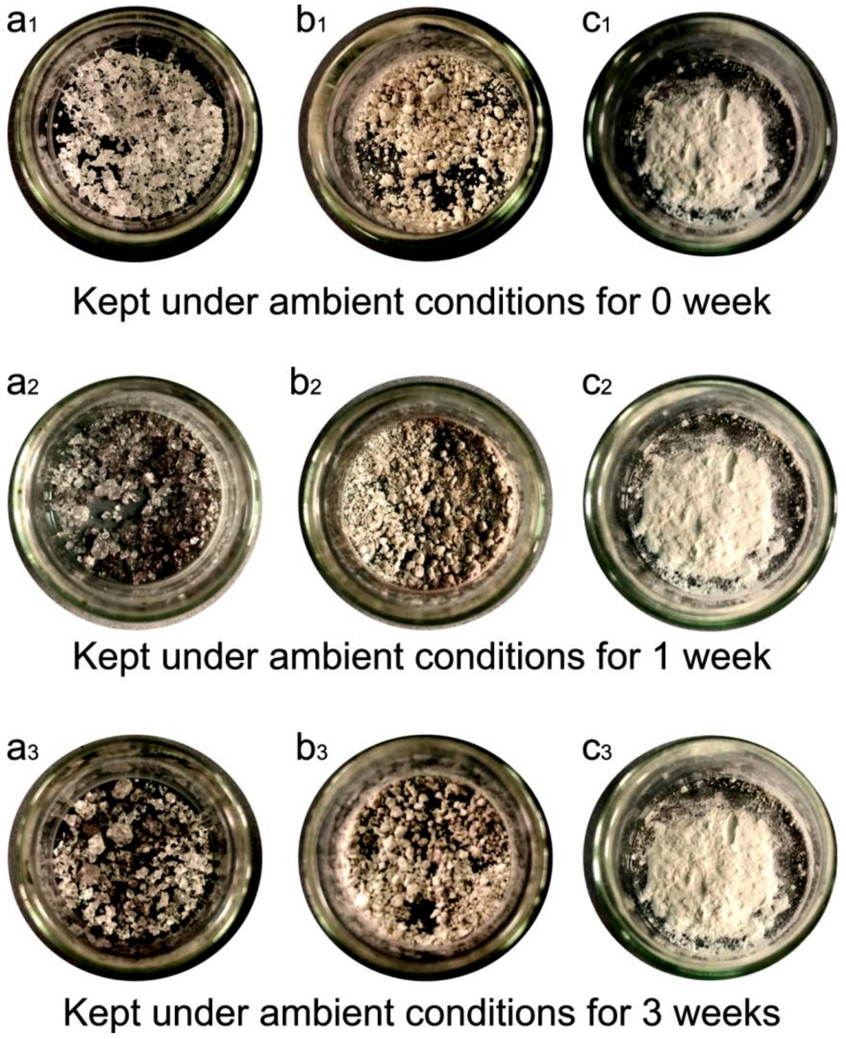

Figure S23 Optical images of $AgNO_3$ (a), CH_3COOAg (b) and Ag_3pz_3 (c) powder. The powders were kept at ambient temperature under an incandescent light.

Table S5 C_3H_6/C_3H_8 separation performance of comparative non-facilitated membranes.

	Membrane	C_3H_6 Permeability (Barrer)	Selectivity	Ref.
Polymer membranes	6FDA-mPD	0.13	10	(*17*)
	6FDA-IpDA	0.58	15	
	6FDA-TrMPD	30	11	
	6FDA-6FpDA	0.89	16	
	6FDA-DDBT	1.8	20	(*18*)
	6FDA-ODA	0.48	11	(*19*)
	PPO	2.3	9.1	
	PDMS	6600	1.1	
	PTPES	181.5^c	184	(*20*)
	PPSU	24.6^c	24.9	
	PSF	113.8^c	1.0	
	PES	13.2^c	2.1	
Mixed-matrix membranes	ZncPIM	837	10.4	(*21*)
	AgcPIM	759	11.7	
	MgcPIM	568	15.2	
	Zr-fum-fcu-MOF/6FDA-DAM	40.4	14.5	(*22*)
	ZIF-8/6FDA-DAM	56.2	31	(*23*)
	ZIF-8/MFI MMM	548	146	(*24*)
	ZC/6FDA-durene	118	18	(*25*)
	EC MMMs	89.9	10.4	(*26*)
	**Ag_3pz_3/PIM-1**	**306**	**194**	**This work**

Table S6 C_3H_6/C_3H_8 separation performance for various membrane operation stability.

Polymer	Filler loading (%)	Operation time (days)	Selectivity	C_3H_6 permeance (GPU)	Ref.
PVP	$AgBF_4$/DOP 50.0[a*]/2.0[b*]	4.2	160	7.5	(*27*)
PVP	$AgBF_4$/DPP 50.0[a*]/2.0[b*]	4.2	135	10	(*27*)
PVP	$AgBF_4$/DPP 50.0[a*]/2.0[b*]	4.2	85	9	(*27*)
PVP	$AgBF_4$/8G1 49.8/0.5[a]	30	50	34	(*28*)
PVP	$AgBF_4$/8G1 49.9/0.2[a]	30	60	34	(*28*)
PEP	$AgNO_3$ 50.0[a]	7	16.2	5.4	(*29*)
POZ	$AgBF_4/Al(NO_3)_3{\cdot}9H_2O$ 47.6/4.8[a]	14	21	4.8	(*30*)
PEO	$AgBF_4/Al(NO_3)_3{\cdot}9H_2O$ 49.9/0.2[a]	10	10	20	(*31*)
PVP	$AgCF_3SO_3/Al(NO_3)_3{\cdot}9H_2O$ 49.9/0.2[a]	4	5	0.5	(*32*)
PVP	$AgCF_3SO_3/Al(NO_3)_3{\cdot}9H_2O$ /$BMImBF_4$	4	9	0.5	(*33*)
PVA	$AgBF_4/Al(NO_3)_3{\cdot}9H_2O$ 49.3/1.5[a]	6	17	11	(*34*)

Table S7 The facilitated transport membrane with metal nanoparticles as carriers for C_3H_6/C_3H_8 separation.

Polymer	Filler loading(%)	Operation time (days)	Selectivity	C_3H_6 permeance (GPU)	Ref.
EPR	Ag NP/p-BQ 35.1/29.8[b]	3.5	11	0.45	(*35*)
SLM*	Ag NP/ $BMImBF_4$ 41.2/58.8[b]	5	17	2.7	(*36*)
POZ	Ag NP/PVP 29.8/35.1[b]	15	21	1.3	(*37, 38*)
PVP	Ag NP/TCNQ 33.1/0.7[b]	5.5	50	3.5	(*39*)
PVP	Ag NP/TTF 33.3/33.3[a]	-	145	2.5	(*40*)
PEO	Ag NP/p-BQ 27.8/2.8[a]	10	10	15	(*41*)
PVP	Ag NP/F4-TCNQ 48.8/2.4[b]	1.5	112	1.8	(*42*)
PU	Ag NP/(OTf) 21.0/29.1[b]	-	24.4[c]	-	(*43*)
**PIM-1**	**Ag_3pz_3**	**20**	**161[d]**	**3.9**	**This work**

Table S8 Performance of C_3H_6/C_3H_8 membrane separation with Ionic liquids to prevent Ag^+ reduction.

Polymer	Filler loading(%)[a]	Operation time (days)	Selectivity	C_3H_6 permeance (GPU)	Ref.
POZ	$AgNO_3$/BMImNO$_3$ 58.8/7.0	6	32	5.6	(*44*)
PVP	$AgBF_4$/BMImNO$_3$ 8.9/86.6	6.5	7.2	3.6	(*45*)
PVDF-HFP	$AgBF_4$/BMImBF$_4$ 60.2/7.8	10	700	55 [e]<br>6630 [f]	(*46*)
PVDF-HFP	BMImBF$_4$/AgBF$_4$	110	144	172 [f]	*(47)*

Tables S6, S7 and S8 legends and abbreviations.

a, mol. %
b, wt. %
a*, mol. % relates only to the polymer
b*, wt. % relates only to the polymer
c, C_3H_6/C_3H_8 ideal solubility selectivity
d, pure gas
e, permeance of C_3H_6 (GPU)
f, C_3H_6 permeability (Barrer).

PEP: poly(ethylene phthalate), PVA: poly(vinyl alcohol), PDMS: polydimethylsiloxane, PVP: polyvinylpyrrolidone, DOP: dioctyl phthalate, DPB: dibutyl phthalate, POZ: poly(2-ethyl-2-oxazoline), POZ: poly(2-ethyl-2-oxazoline), DPP: diphenyl phthalate, 8G1: *n*-octyl β-D-glucopyranoside, PU: polyurethane, Ag NP: silver nanoparticle, PEO: poly(ethylene oxide), p-BQ: p-benzoquinone, BMImBF$_4$: 1-butyl-3-methylimidazolium tetrafluoroborate, TCNQ: 7,7,8,8-tetracyanoquinodimethane, TTF: tetrathiafulvalene, (OTf): triflate anion, PVDF-HFP: poly(vinylidene fluoride-*co*-hexafluoropropylene), F4-TCNQ: 2,3,5,6-tetrafluoro-7,7,8,8-tetracyanoquinodimethane.

REFERENCES AND NOTES:

1. G. M. Sheldrick, SHELXT: *Acta Crystallogr., Sect. A: Found. Adv* 2014, *70*, C1437.
2. O. V. Dolomanov, L. J. Bourhis, R. J. Gildea, J. A. Howard, H. Puschmann, *Journal of Applied Crystallography* 2009, *42,* 339-341.
3. G. M. Sheldrick, Crystal structure refinement with SHELXL. *Acta Crystallographica Section C: Structural Chemistry* 2015, *71*, 3-8.
4. N. Du, G. P. Robertson, J. Song, I. Pinnau, S. Thomas, M. D. Guiver, *Macromolecules* 2008, *41*, 9656-9662.
5. G. Kresse, J. Furthmüller, *Physical Review B* 1996, *54*, 11169.
6. G. Kresse, J. Furthmüller, *Computational Materials Science* 1996, *6*, 15-50.
7. J. P. Perdew, K. Burke, M. Ernzerhof, *Physical Review Letters* 1996, *77*, 3865.
8. P. E. Blöchl, *Physical Review B* 1994, *50*, 17953.
9. H. J. Monkhorst, J. D. Pack, *Physical Review B* 1976, *13*, 5188.
10. M. Methfessel, A. Paxton, *Physical Review B* 1989, *40*, 3616.
11. J. Klimeš, D. R. Bowler, A. Michaelides, *Physical Review B* 2011, *83*, 195131.
12. J. Huang, S. Rauscher, G. Nawrocki, T. Ran, A. D. Mackerell, *Biophysical Journal* 2017, *112,* 175a-176a.
13. M. J. Abraham, T. Murtola, R. Schulz, S. Páll, J. C. Smith, B. Hess, E. Lindahl, *SoftwareX* 2015, *s1–2*, 19-25.
14. K. Vanommeslaegh, E. Hatcher, C. Acharya, S. Kundu, S. Zhong, J. Shim, E. Darian, O. Guvench, P. Lopes, I. Vorobyov, A. D. Mackerell, *Journal of Computational Chemistry* 2010, *31*, 671-690.
15. H. Heinz, T. J. Lin, R. Kishore Mishra, F. S. Emami, *Langmuir* 2013, *29*, 1754-1765.
16. Y. Liu, R. Wang, T. S. Chung, *Journal of Membrane Science* 2001, *189,* 231-239.
17. C. Staudt-Bickel, W. J. Koros, *Journal of Membrane Science* 2000, *170,* 205-214.
18. K. Okamoto, K. Noborio, J. Hao, K. Tanaka, H. Kita, *Journal of Membrane Science* 1997, *134*, 171-179.
19. K. Tanaka, A. Taguchi, J. Hao, H. Kita, K. Okamoto, *Journal of Membrane Science* 1996, *121*, 197-207.
20. C. Liang, W. Yong, J. Wu, M. Weber, C. Maletzko, J.-Y. Lai, T.-S. Chung, *Journal of Membrane Science* 2022, *647*, 120293.
21. K.-S. Liao, J.-Y. Lai, T.-S. Chung, *Journal of Membrane Science* 2016, *515*, 36-44.

22. Y. Liu, Z. Chen, G. Liu, Y. Belmabkhout, K. Adil, M. Eddaoudi, W. Koros, *Advanced Materials*, 2019, *31*, e1807513.
23. C. Zhang, Y. Dai, J. R. Johnson, O. Karvan, W. J. Koros, *Journal of Membrane Science* 2012, *389*, 34-42.
24. F. Rashidi, J. Leisen S. J. Kim, A. A. Rownaghi, C. W. Jones, S. Nair, *Angewandte Chemie International Edition* 2019, *58*, 236-239.
25. R. Lin, L. Ge, H. Diao, V. Rudolph, Z. Zhu, *Journal of Materials Chemistry A* 2016, *4*, 6084-6090.
26. B. Yuan, H. Sun, T. Wang, Y. Xu, P. Li, Y. Kong, Q. J. Niu, *Scientific Reports* 2016, *6*, 28509.
27. B. Jose, J. H. Ryu, B. G. Lee, H. Lee, Y. S. Kang, H. S. Kim, *Chemical Communications*, 2001, *20*, 2046-2047.
28. H. H. Park, J. Won, S.-G. Oh, Y. S. Kang, *Journal of Membrane Science* 2003, *217*, 285-293.
29. S. W. Kang, J. H. Kim, K. Char, Y. S. Kang, *Industrial & Engineering Chemistry Research* 2006, *45*, 4011-4014.
30. S. W. Kang, J. H. Kim, J. Won, Y. S. Kang, *Journal of Membrane Science* 2013, *445*, 156-159.
31. D. Song, Y. S. Kang, S. W. Kang, *Journal of Membrane Science* 2015, *474,* 273-276.
32. Y. S. Park, Y. S. Kang, S. W. Kang, *Journal of Membrane Science* 2015, *495,* 61-64.
33. Y. S. Park, S. W. Kang, *Chemical Engineering Journal* 2016, *306*, 973-977.
34. Y. S. Park, S. Chun, Y. S. Kang, S. W. Kang, *Separation and Purification Technology* 2017, *174*, 39-43.
35. Y. S. Kang, S. W. Kang, H. Kim, J. H. Kim, J. Won, C. K. Kim, K. Char, *Advanced Materials* 2007, *19*, 475-479.
36. S. W. Kang, K. Char, Y. S. Kang, *Chemistry of Materials* 2008, *20*, 1308-1311.
37. S. W. Kang, Y. S. Kang, *Journal of Colloid and Interface Science* 2011, *353*, 83-86.
38. H. S. Shin, H. J. Yang, S. B. Kim, M. S. Lee, *Journal of Colloid and Interface Science* 2004, *274*, 89-94.
39. I. S. Chae, S. W. Kang, J. Y. Park, Y. G. Lee, J. H. Lee, J. Won, Y. S. Kang, *Angewandte Chemie International Edition* 2011, *50*, 2982-2985.
40. H. Cho, H. Choi, J. H. Lee, Y. R. Kim, D. Song, S. W. Kang, S. S. Lee, Y. S. Kang, *Chemical Communications* 2014, *50*, 3194-3196.
41. G. H. Hong, D. Song, I. S. Chae, J. H. Oh, S. W. Kang, *RSC Advances* 2014, *4*, 4905-

4908.

42. I. S. Chae, S. W. Kang, Y. S. Kang, *RSC Advances* 2014, *4*, 30156-30161.
43. C. G. F. Rezende, C. P. Borges, A. C. Habert, *Journal of Applied Polymer Science* 2016, *133*, 42916.
44. S. W. Kang, K. Char, J. H. Kim, C. K. Kim, Y. S. Kang, *Chemistry of Materials* 2006, *18*, 1789-1794.
45. S. W. Kang, K. Char, J. H. Kim, Y. S. Kang, *Macromolecular Research* 2007, *15*, 167-172.
46. M. Fallanza, A. Ortiz, D. Gorri, I. Ortiz, *Journal of Membrane Science* 2013, *444*, 164-172.
47. Zarca R, Campos A C C, Ortiz A, Gorria D., Ortizet I. *Journal of Membrane Science* 2019, *572*, 255-261.
